# GDTN: Genome-Based Delay Tolerant Network Formation in Heterogeneous 5G Using Inter-UA Collaboration

**DOI:** 10.1371/journal.pone.0167913

**Published:** 2016-12-14

**Authors:** Ilsun You, Vishal Sharma, Mohammed Atiquzzaman, Kim-Kwang Raymond Choo

**Affiliations:** 1 Department of Information Security Engineering, Soonchunhyang University, Asan-si 31538, Republic of Korea; 2 Computer Science and Engineering Department, Thapar University, Patiala, Punjab, India, 147004; 3 School of Computer Science, University of Oklahoma, Norman, OK, United States of America; 4 Department of Information Systems and Cyber Security, The University of Texas at San Antonio, San Antonio, TX 78249 United States of America; Semmelweis University, HUNGARY

## Abstract

With a more Internet-savvy and sophisticated user base, there are more demands for interactive applications and services. However, it is a challenge for existing radio access networks (e.g. 3G and 4G) to cope with the increasingly demanding requirements such as higher data rates and wider coverage area. One potential solution is the inter-collaborative deployment of multiple radio devices in a 5G setting designed to meet exacting user demands, and facilitate the high data rate requirements in the underlying networks. These heterogeneous 5G networks can readily resolve the data rate and coverage challenges. Networks established using the hybridization of existing networks have diverse military and civilian applications. However, there are inherent limitations in such networks such as irregular breakdown, node failures, and halts during speed transmissions. In recent years, there have been attempts to integrate heterogeneous 5G networks with existing ad hoc networks to provide a robust solution for delay-tolerant transmissions in the form of packet switched networks. However, continuous connectivity is still required in these networks, in order to efficiently regulate the flow to allow the formation of a robust network. Therefore, in this paper, we present a novel network formation consisting of nodes from different network maneuvered by Unmanned Aircraft (UA). The proposed model utilizes the features of a biological aspect of genomes and forms a delay tolerant network with existing network models. This allows us to provide continuous and robust connectivity. We then demonstrate that the proposed network model has an efficient data delivery, lower overheads and lesser delays with high convergence rate in comparison to existing approaches, based on evaluations in both real-time testbed and simulation environment.

## 1 Introduction

With multiple radio access networks operating in different network units, it is of utmost importance to be able to utilize the entire resources in such networks. The current trend is to deploy 5G network with different configuration-based radios operating in coordination in order to provide a user with better connectivity and data rate (i.e. increase user’s quality of experience). Multi-channel diversity needs to be addressed in order to ensure optimum utilization of network resources. Resource and data management are two primary tasks in these collaborative radio networks. However, effectively achieving these tasks is complicated by networks operating with multiple applications (having varying demands in data rates) and different running facility [1, 2]. The challenges are compounded as the user base becomes more Internet-savvy and sophisticated and require a wider coverage. Thus, hybridization is identified as a possible solution to improve coverage without increasing the overheads.

Hybrid networking using independent networks has a wide range of applications in both military and civilian activities [3], as these networks can provide robust connectivity to achieve complex tasks efficiently over 5G networks. Current hybrid formations over 5G networks may comprise static, ground-mobile, or aerial nodes. How to efficiently achieve diversity in node configuration and continuous connectivity in these networks is of ongoing research interests, and one of the applications using these multiple networks in the form of 5G is the formation of delay tolerant networks (DTNs). DTNs do not require an end-to-end connectivity during the transfer of data and are capable of tolerating some data loss.

In DTNs, routes are found over multiple unreliable hops which are connected intermittently. These networks have been the subject of research in recent years. Hybrid networks comprise nodes with different types, makes, and configurations and routing is one of the main aspects of such networks [4]. Efficient routing approaches are known to enhance the applicability of DTNs. For example, multi-hop routing can be used to provide continuous connectivity [5]. We observe that existing solutions for DTNs primarily focus only on the routing aspect, but not on the topological arrangement and initial network configurations [6].

Hybrid networks involve applications that can be operated in scenarios with more overheads than regular DTNs. One possible hybrid DTN formation is the use of Unmanned Aircraft (UA) as network nodes, where UA serves the purpose of relays during transmission and is capable of providing a strong inter-connectivity between the ground nodes. However, these types of UA-networks require ongoing support from the network components so as to form a fully-reliable and a fault-tolerant network.

Major challenges faced by hybrid networks include irregular breakdown, node failures, and halts during fast transmissions. In recent years, there have been attempts to integrate hybrid and ad hoc networks (i.e. delay-tolerant hybrid networks) in the form of packet switched networks, with the aims of providing a robust solution [3] [7]. However, to the best of our knowledge, there is no known approach that provides a consistent and accurate solution to inter-networking between the ground and the aerial nodes. Existing state-of-the-art DTN models include Bubble [7], Epidemic [8], Prophet [9] and greedy forwarding. These approaches are suitable only for static networks with deterministic states. However, for networks operating in highly dynamic environments, these models are not able to cope with the network faults and delays. This is the gap we seek to address in this paper. Specifically, we seek to improve the coordination between the ground nodes by allowing connectivity via UA which acts as on-demand relays. The inter-UA network allows efficient data flow with reduced overheads and improved convergence rate, in comparison to existing approaches. Also, the proposed approach aims to provide a better packet delivery ratio even in the case of high randomness with lower overheads, reduced delays, and higher convergence rate.

In this paper, we present a novel model for delay tolerant network formation, which uses multiple UAs as a pivot network between existing hybrid connections. Our model is based on a genome-based approach, which efficiently maps the nodes of different networks and identifies whether relaying is required in the intra- and inter-network environment. The proposed approach is bio-inspired and derives from modern molecular biology. The genome-based approach allows the formation of natural computation which can be readily applied to the problems of network mapping and communications. The genome is the collection of various biological material which combines together to form the entire genetic material. Similar to this, the networks with varied nodes can be equalized with the genomes with various devices and components acting as its genetic material. Further, the genetic material of the genomes is mapped with respect to their properties and utility with high accuracy. The similar pattern can be used in the formation of the delay tolerant network, which allows network components to be mapped with high accuracy. The genetic material also forms the basis for the properties of a genome; similar to this, the network genetic material (devices and components) forms the basis for the properties of a delay tolerant network. Thus, all these similarities provide the ideology for choosing genome-based networks as the basic functional unit behind the proposed approach.

In the proposed approach, the network nodes are treated as chromosomes, which are then genetically mutated to form a complete genome. This genome replicates the complete network model and enhances the UA coordinated network formation. Further, repetitive and non-repetitive DNA formation is used to combine the nodes from different networks. The proposed approach efficiently incorporates the use of UA to form a continuous and robust delay tolerant network. The evaluations demonstrate that the proposed approach provides better connectivity in DTNs with improved data rate. The proposed approach outperforms existing approaches in delivery rate and overheads even in the case of node failures. It also overcomes the limiting applicability of approaches in Bubble [7], Epidemic [8], Prophet [9] and greedy forwarding by not only providing efficient connectivity in a static network with deterministic states but also in highly dynamic networks with unknown failures and traffic demands.

### 1.1 Problem Description and Our Contribution

The problem deals with the formation of an efficient delay tolerant network using UAs as pivotal nodes for the entire network. The selection of the intra- and inter-relays for efficient data forwarding using these UAs is the primary task. The problem is to optimally identify these relays and select the appropriate route which is capable of sustaining the network failures, breakdowns, and delays. Optimized data flow with minimal delay and fewer overheads is the major aspect of the considered network. Further, the problem also deals with the mapping of the nodes with the UAs using the concept of genomes. This problem is subjected to the identification of appropriate devices and components which will act as the genetic material for the considered network model. The contributions of the proposed work are as follows:

- The proposed model provides a novel inter-UA collaboration for efficient connectivity in the DTNs.
- The proposed network formation provides a UA pivot between the hybrid networks for forming a network that can withstand uncertain network demands, high traffic rate, and irregular network breakdowns.
- The proposed model provides better flow in highly dynamic UA networks even in the case of high randomness with lower overheads, lesser delays, and high convergence rate.

The rest of the paper is structured as follows: Section II briefly reviews existing literature on DTNs. Section III discusses genomes and presents the proposed model and the underlying building blocks. Evaluations are described in Section IV. Section V presents a comparative summary, discussions, and open issues. Finally, Section VI concludes the paper.

## 2 Related Work

DTNs aim at resolving continuous connectivity issues in hybrid networks. These networks focus on network formations in extreme dynamic conditions, or in the case of highly mobile scenarios. A number of models have been proposed for the formation of efficient DTNs, such as GeoSpray by Soares et al. [10] designed for vehicular networks. This model uses geographically collected location information when making routing decisions. Jones et al. [5] also developed a routing protocol for DTNs, by utilizing the average waiting time for the next hop to distribute traffic in the network using a link state routing protocol.

Gil-Castiñeira et al. [11] demonstrate that delay tolerant vehicular ad hoc network is more reliable than a simple vehicular ad hoc network by proposing and analyzing the extension of vehicular controller area field buses. In their approach, they utilize the multihop rather than the single hop vehicle-to-vehicle paradigm. The distributed scheme of Chen et al. [12] is based on the estimation and collaborative control for industrial systems using wireless sensor-actuator networks. The social-based forwarding protocol, BUBBLE, of Hui et al. [7] is designed to enhance the delivery probability by utilizing two social and structural metrics with real mobility traces of human. The approach of Han et al. [6] leverages opportunistic communications in emerging mobile social networks to facilitate information dissemination and reduce mobile data traffic.

Rubinstein et al. [13] explain that in delay tolerant vehicular networks, when cars travel at 60km/hr with duration of contact of 11s, only about 80 KB of data is transferred using TCP connection and UDP outperforms TCP by transferring 2MB of data at that point of contact. Thus, vehicular delay-tolerant networks can be used to route larger-size packets instead of small packets [4]; consequently, requiring less packet processing with reduced complexity. Pereira et al. [14] focus on routing issues in vehicular delay tolerant networks with huge variations in density, while AAkerberg et al. [15] focus on delay variations in wireless sensor networks for process automation in industry.

Shuai et al. [16] study the motion control of autonomous vehicles with respect to the onboard time delays induced by the network providing robustness and better control against varying network delays. Rao et al. [17] design a solution, *Ameba*, for the timely delivery of the messages in DTNs using content properties, hop count, locations, and interest of nodes. Khanesar et al. [18] seek to address packet loss and network-induced delays by proposing a fuzzy based sliding mode controller, which employs the fuzzy system to estimate the nonlinear dynamic system. Schoeneich et al. [19] develop an approach for the indoor application of drones with delay tolerant network formations. Their approach is expandable for use in emergency conditions.

Tian et al. [20] develop a three-dimensional location based protocol 3DLEAR, which uses the 3D location information of nodes for routing of real delay tolerant networks. Yu et al. [21] develop a hybrid routing algorithm based on probabilistic data delivery and redundancy to reduce the network overheads. You et al. [22] develop a movement pattern-aware optimal routing (MPAR) with its application specifically on the social delay tolerant networks. The protocol uses the local and tabu-search scheme to determine the optimized path between the source and the destination over the nodes with social behaviour. Stewart et al. [23] develop a congestion avoidance shortest path routing (CASPaR) to maximize the packet delivery ratio with minimal latency in delay tolerant networks. Burns et al. [24] introduce a multi-objective robotic assistance routing (MORA) for disruption tolerant networks capable of generating optimized movements. Spray and Wait (SAW) and MaxProp are the other popular protocols for delay tolerant networks, developed by Spyropoulos et al. [25] and Burgess et al. [26], which aims at link improvements of nodes.

All the above-discussed approaches aims at routing in most of the time and do not account for network topology and node arrangements. Further, these approaches are unable to tackle the irregular breakdowns, node failures, and halts during fast transmissions. Also, these models are yet to be improved for their direct applicability on the UA-assisted delay tolerant networks. Thus, we remark that cooperative UA can provide better control and connectivity in wireless networks (see [3, 27]), and networking with UA can resolve issues relating to the availability of continuous links by providing opportunistic relaying when required (see [28, 29]).

## 3 Proposed Network Formation

**Fig 1:**
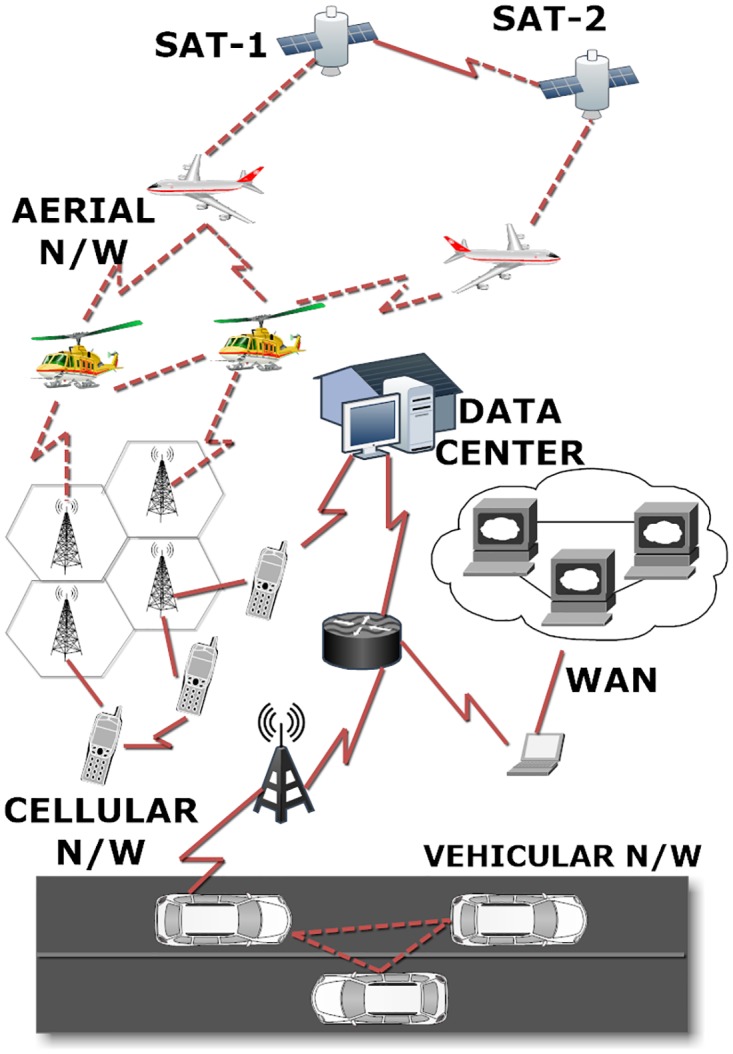
Hybrid Delay Tolerant Network.

The proposed multiple network collaboration converges towards the formation of a delay tolerant coordinating unit that operates over underlying network components. Extracting the features of the existing network, and then utilizing them for a semi-major network formation is the primary objective of this approach. “Semi” means that only capable nodes in the network are allowed to interact, whereas “major” defines that the underlying networks operate as a traditional unit while the above network operates as the major corresponding network. The proposed network derives its features from the biological combining of creatures to produce new species. Similarly, multiple networks are combined to form a conceptually-virtual network which possesses different features as genetics from different networks, as shown in Fig 1. These genetic-oriented features distinguish the networks combined together to relay data.

In the proposed model, the nodes of the same network will have a similar genetic composition. Based on this genetic ideology, the proposed model is termed the Genome-based Delay Tolerant Network formation (GDTN). Use of UA in these networks allows a strong interconnection which is capable of attaining a zero-delay aspect while drifting data from one network to another. UA plays the role of cross-mutator that allows interaction between different genome characteristic networks. In order to better understand the concept of the proposed model, the ideology of the biological aspects operating behind the whole network are discussed next.

### 3.1 Genome Structure

The idea behind the formation of the hybrid network with the provision of delay tolerance is motivated from the requirement of inter-connectivity between different networks. This allows us to provide a strongly connected network that can efficiently relay information in real-time, without geographical constraints. Combining multiple networks to form a virtual independent network is similar to the biological aspects of living creatures that derive their characteristics from the genetics mutated from their parents. The genome is the main identifier of an object which possesses sign of life [30].

These genomes bear the coding and non-coding based schema divided into two units, namely: DNA and RNA. These genomes comprise chromosomes which further collaborate to form a gamete or genome. Using the genomes as the main ideology behind the formation of the proposed delay tolerant hybridization, the genome features have been extended for their application to wireless networks. Two major features, core genetic material, and additional genetic material, are used to classify the network formation. For the core genetic material, the following characteristics are used:

- Genome Size (*N*_*s*_): genome size replicates the network size denoting number of nodes in a network.
- Repetitive DNA (*N*_*r*_): random nodes which are non-coded and does not follow any rules.
- Non-repetitive DNA (*N*_*f*_): nodes fixed in terms of configurations which can be controlled are considered as non-repetitive DNA.
- Chromosomes (*N*): it denotes the network node.
- Chromosome Number (*I*_*n*_): unique identification allotted to a node.
- Chromosome Type (*N*_*t*_): network-id to which a node belongs.
- Gene Order (*S*_*q*_): sequence number for collaboration.
- Gene Content (*G*_*c*_): Boolean counter which identifies the collaboration and count for each success/failure of inter-connections.

For additional genetic materials, the following characteristics are used in the network model:

- Antenna Type (*A*_*t*_): Radio transmitter type.
- Bandwidth (*B*): range of frequencies supported by the network.
- Radio Range (*R*_*r*_): radio range of network nodes.
- Link Type (*L*_*t*_): link between the network nodes.
- Traffic Rate (*T*_*r*_): transmission speed based on the link.

Core genetic materials defined for the network form the history of the network, which is used to idealize the state of the network in the event of failures or re-caching of the stabilized state. Further, the chromosome types *N*_*t*_ are used to identify whether the connection is intra- or inter-state. For intra-state connections, sender and receiver have the same value for *N*_*t*_; whereas for inter-state connectivity, both sender and receiver have different values for *N*_*t*_.

### 3.2 Network Ideology

Collaborative network aims at formation of a set *X*, which comprises networks with different genome size and genome order (i.e. *X* = {*G*_1_, *G*_2_…*G*_*n*_}, where *n* defines the number of networks operating in coordination, and *G* bears characteristics of wireless network and satisfies all properties of genome-based network formations). Such network formation should have a minimum delay in gene mapping and sequencing. Gene mapping is the term used for identification of network nodes for relaying, and sequencing determines the order of mapping. Lower delays in the two properties allow the formation of an efficient delay-tolerant network. The main properties of the network formed by the aspect of mapping and sequencing are as follows:

- For better identification of the gene order, no more than two nodes are allowed to collaborate on the same channel as this will prevent mutations that may hinder the network performance.
- Network with least genome size will act only as helper network and will not directly collaborate with any another network on its own. This will prevent resources and will help to attain continuous connectivity.
- Sharing of genome tables which is an alternative to routing information table in the proposed approach will be done between the mapped networks only. This will prevent any anti-security actions of the nodes.
- Nodes will advertise their collaboration and mapping information but will not advertise the frequency channel or the information about the collaborating node. This will prevent possible middle attacks that may interrupt the network by causing delays. All mapping information will be shared as boolean only.
- Each network will create a log, which can be shared between the dominating or any node in the collaborative network. This will help to trace the network activity and cache the network history for information retrieval and debug when required.

In the proposed model, the mapping between the intra- and inter-network nodes is carried using the Gauss-Markov [31], and the *α*-*β*-*γ* filter [32]. Both these models follow a decay principle that can be treated similarly with the mutations carried out by the chromosomes. These models allow the formation of the system model with respect to the usage of network components that allow correct node mapping in a hybrid environment, and also provide a solution for failure prediction in hybrid delay tolerant environments. In the proposed model, *F*_1_ and *F*_2_ are considered to be the mapping function for the complete network fairness in the intra- and inter-mode, respectively, such that

$$F_{1}=f\left(N_{s},N_{r},N_{f},G_{c}\right),$$ (1)

and

$$F_{2}=f\left(I_{n},N_{t},S_{q},G_{c}\right).$$ (2)

Now, for the defined mapping in Eqs (1) and (2), *F*_1_ and *F*_2_, at any time instance *t*, can be projected using the Gauss-Markov model as

$$F_{1}^{t}=\alpha_{1}F_{1,0}+\alpha_{2}F_{1}^{{}^{\prime \prime }}+\alpha_{3}F_{1,R}+\epsilon_{1},$$ (3)

where *α*_1_, *α*_2_, and *α*_3_ are the decay constants for selected chromosomes derived using *α*-*β*-*γ* filter [32], such that 0 ≤ *α*_1_ ≤ 1,

$$\alpha_{2}=\alpha_{1}e^{-\alpha_{1}},$$ (4)

$$\alpha_{3}=\frac{\alpha_{1}}{\alpha_{2}+\alpha_{1}},$$ (5)

and

$$F_{1}^{{}^{\prime \prime }}=\left[\left(\begin{array}{c} N_{s}\\ N_{r}\\ N_{f}\\ G_{c} \end{array}\right)\right].$$ (6)

Here, *F*_1,0_ denotes the initial value for the mapping function *F*_1_. For this intra-network connectivity, *F*_1,*R*_ is the Gaussian estimator for fairness such that

$$F_{1,R}=\left(\frac{\sum_{i=1}^{N_{s}}F_{1,0}F_{1,i}}{\sum_{i=1}^{N_{s}}\frac{F_{1,i}^{2}}{\sum_{j=1,j\neq i}^{N_{s}}F_{i,j}}}\right),$$ (7)

and

$$\epsilon_{1}=F_{1,R}\left(\sum_{i=1}^{N_{s}}F_{1,i}\left(e^{-\left(\frac{\alpha_{3}}{\alpha_{1}+\alpha_{2}}\right)}\right)\right).$$ (8)

Similarly, for inter-network mapping, *F*_2_, at time instance *t*, is computed as

$$F_{2}^{t}=\alpha_{1}F_{2,0}+\alpha_{2}F_{2}^{{}^{\prime \prime }}+\alpha_{3}F_{2,R}+\epsilon_{2},$$ (9)

and

$$F_{2}^{{}^{\prime \prime }}=\left[\left(\begin{array}{c} I_{n}\\ N_{t}\\ S_{q}\\ G_{c} \end{array}\right)\right].$$ (10)

Here, *F*_2,0_ denotes the initial value for the mapping function *F*_2_. Similar to intra-network formations, *F*_2,*R*_ is the Gaussian estimator for fairness over inter-network formations such that

$$F_{2,R}=\left(\frac{\sum_{i=1}^{K}F_{2,0}F_{2,i}}{\sum_{i=1}^{K}\frac{F_{2,i}^{2}}{\sum_{j=1,j\neq i}^{K}F_{i,j}}}\right).$$ (11)

Here, *K* is the maximum count for *S*_*q*_, and

$$\epsilon_{2}=F_{2,R}\left(\sum_{i=1}^{K}F_{2,i}\left(e^{-\left(\frac{\alpha_{3}}{\alpha_{1}+\alpha_{2}}\right)}\right)\right).$$ (12)

Using *F*_1_ and *F*_2_, the network fairness $\left(S_{F_{1},F_{2}}^{t}\right)$ which marks the efficient utilization of network resources and accurate mapping is calculated as

$$S_{F_{1},F_{2}}^{t}=\left|\left(\sum_{i=1}^{N_{s}}\left(\sum_{j=1}^{K}F_{2,j}\right)F_{1,i}\right)E\left(\frac{F_{1}^{t}}{F_{2}^{t}}\right)\right|.$$ (13)

The system fairness in Eq (13) is used to trace the accurate mapping of the network nodes with respect to the threshold mapping, which is computed using Jain Fairness Index [33] as

$$S_{F}^{TH}=\frac{\sum_{i=1}^{N_{s}}F_{1,R,i}}{N_{s}\;\sum_{j=1}^{K}F_{2,R,j}}.$$ (14)

At any instance in the network, efficient connectivity can be traced iff $S_{F_{1},F_{2}}^{t}$ ≥ $S_{F}^{TH}$.

In a network, the initial condition of dissatisfaction of Eq (14) predicts the possibility of connection failure. For prediction of the induced failures in the intra-network formations, the following mapping function is used to trace the failure occurrence.

$$F_{3}=\left(N_{r},N_{f}\right)$$ (15)

For an induced failure, *N*_*r*_ > >*N*_*f*_, i.e. with more non-coded nodes in the networks, there are more chances of the occurrence of failures. Similarly, for inter-network formations, an account of the sequence count *S*_*q*_ allows prediction of the failures in the network that may increase the delay beyond the limiting value. Thus, in inter-network formations, an induced failure is accounted if $N_{r}\geq \frac{S_{q}}{k}$, where *k* is the limiting count for the network to be in the inactive state. In the proposed model, *k* = 2, i.e. at least half of the network nodes should be accurately connected.

### 3.3 Delay Model

A network with multiple sub-divisions which are a complete network in themselves cannot eradicate delay effect. Also, some of sought of jitters are bound to happen when a network address changes or when a node drops during inter-network operations. But still, by understanding the concept of delay affecting parameters in variedly operating networks, these can be minimized to form a robust-delay tolerant network.

For the proposed network formation, delays are identified over genome structure of the network. General formulation for the delays is considered which comprise genome processing delay *G*_*prd*_, genome transmission delay *G*_*td*_, genome queuing delay *G*_*qd*_, and genome propagational delay *G*_*pd*_. These delays sum up to compute the overall delay for the network. Processing and queuing delays denote the time lapsed in mapping the network nodes to form a final network with connectivity between the source and the destination. Propagational and transmission delays denote the sequencing time and transfer time of data between the source and the destination. Processing delay and queuing delay are given as delay time function *D*_*A*_ such that:

$$D_{A}=\int_{N_{s}}\left(G_{prd}+G_{q}\right)dt$$ (16)

where

$$G_{prd}=\tau_{1}\left(N_{s}-N_{r}\right),$$ (17)

and

$$G_{q}=\tau_{2}\left(N_{R}\right)$$ (18)

Here, *τ*_1_ and *τ*_2_ denote the minimum time required in mapping actual configured nodes and time required to identify the random nodes, respectively. Also, *G*_*pd*_ is computed over the received signal strength *R*_*ss*_ with respect to the nodes connected over the inter-network formation as:

$$G_{pd}=\frac{C_{h}}{\sqrt{R_{ss}}\times speed}$$ (19)

where *R*_*ss*_ is derived from [34] as:

$$R_{ss}=Pow_{min}-Avg_{\eta }\;\;\eta \;log_{10}\;\;\left(\frac{D}{R_{b}}\right)$$ (20)

where *Pow*_*min*_ is the minimum power strength available in the signal, *Avg*_*η*_ is the average path loss, *η* is the path loss, *D* is the network range and *R*_*b*_ is the radius up to which prescribed bandwidth is supported. *C*_*h*_ is the chromosome constant computed as:

$$C_{h}=\frac{\tau_{1}+\tau_{2}}{2\;\eta }\left(\frac{N_{f}-Relays}{N_{r}}\right)$$ (21)

Transmission delays depend on the successful delivery of data between the network nodes and the link speed available for transmission.

**Remarks:** Chromosome constant identifies the correct pairing of network nodes for relaying based on the coding. Coding refers to configurable nodes. The higher the value of chromosome constant, the lower will be the randomness and the higher will be the coding; thus, resulting in reduced network delays.

### 3.4 Effect of Randomness

The lower the randomness, the higher is the degree of connectivity; thus, achieving a more stable network. With lower randomness, as suggested in the biological description of genome formation [30], the degree of coding increases; thus, the number of configurable and controllable nodes also increases. According to the properties of a genome, randomness increases with increases in distance. As distance increases, the number of participant nodes available for connectivity decreases. Thus, selection of optimal distance for a better genetic mutation in such networks is an optimization problem in itself. However, this paper does not deal with the geometrical distance aspect of the nodes; rather, it operates in selection of optimum nodes required for the formation of delay tolerant inter-network.

***Lemma—1***: *With a consistent increase in genome size, properties of DNA varies, randomness increases and the number of non-repetitive units decreases. Similar effect is observed in genome-based networks (i.e. with a consistent increase in network area, distance and network size, randomness increases); thus, resulting in delays increase.*

***Proof:*** Let *Dis*_*th*_ be the threshold distance which is required to be maintained for continuous connectivity, *G*_*th*_ be the genome size representing the network size up to which the network is controllable, *D*_*th*_ be the limiting values of delay beyond which the network does not respond. Using definition of received signal strength *R*_*ss*_ from Eq (20),

$$R_{ss}\propto -\left(Dis\right).$$ (22)

Thus, with increase in distance (*Dis* > >*Dis*_*th*_, *N*_*s*_ > >*G*_*th*_), received signal strength of the network decreases. Also, *R*_*ss*_ is much affected by the path loss constant for the network. Using the definition of path loss [34], with more randomized nodes, the path loss increases and adversely affects the network. From Eq (20), increasing value of path loss decreases the *R*_*ss*_. From Eq (19), a decrease in *R*_*ss*_, increases the propagational delays which are the dominating factor in computing the overall network delay. Thus, it can be concluded that with an increase in network size and randomness, network delay increases beyond the threshold.

***Proposition-1:*** From the Lemma-1, it can be deduced that for the complete connectivity between the nodes i.e. for the formation of connected graph, degree of randomness should be minimum.

***Lemma—2:*** *In a network formed using the properties of genome-based model, network stability increases with decrease in randomness and network size/genome size.*

***Prerequisite:*** Before proof, it is to be noted that this lemma is applicable to the networks which utilize the properties of the genome-based networks during the intra- and inter-network communications. However, for its generic applicability to the other network models, the network structure or topology is required to be analyzed beforehand to accurately analyze the effect of randomness.

**Fig 2:**
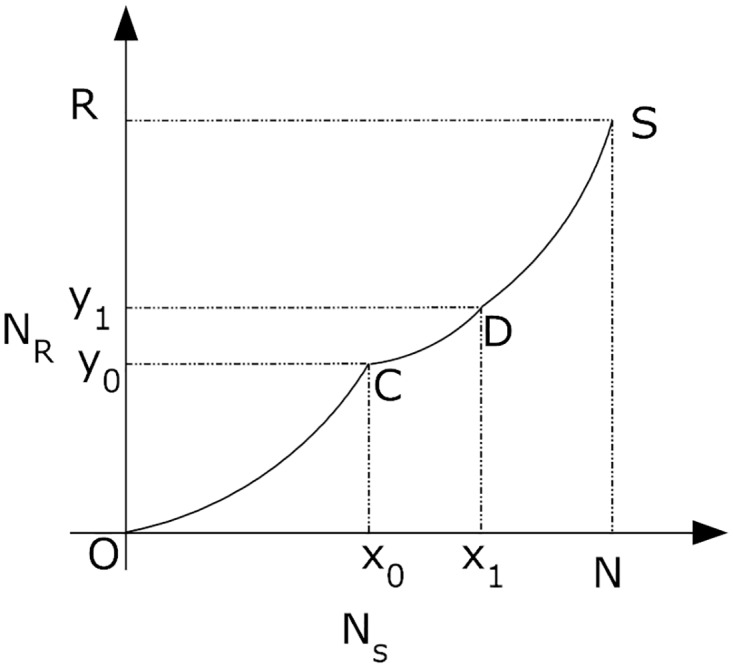
Monotonically Increasing Network Instability Curve.

***Proof:*** In the proposed approach, this lemma is proved using inverse stability law which defines that the network instability *N*_*is*_ is the function of randomness *N*_*r*_ and network size *N*_*s*_. From the lemma-1, and proposition-1 it can be deduced that the network instability increases as the network randomness increases and attains maxima if both randomness and network size consistently increases. This sort of pattern is exhibited by a monotonically increasing curve as shown in Fig 2. In Fig 2, curve CD denotes the constant delay which does not affect the performance of the network. It is a non-decreasing value which has delays controlled under certain limit, thus, preventing any hinderance in network operations. As a function of randomness and network size, network instability is the area under curve presented in Fig 2. For a defined network, at point *x*_0_,

$$N_{r}^{{}^{\prime }}\propto e^{x_{0}}$$ (23)

$$N_{r}^{{}^{\prime }}\propto \left(x_{0}-\frac{x_{0}}{G_{c}\;M_{p}}\right)$$ (24)

where *M*_*p*_ is the mapped nodes with genome content *G*_*c*_. Using slope of curve *C*′ as randomness constant, randomness can be defined as function of network size such that:

$$N_{r}=C^{{}^{\prime }}\;N_{s}\;e^{N_{s}}\left(1-\frac{1}{G_{c}\;M_{p}}\right)$$ (25)

Network instability *N*_*is*_ is defined as area under curve plotted between randomness *N*_*r*_ and network size *N*_*s*_. Therefore,

$$N_{is}=\left(1-\frac{1}{G_{c}\;M_{p}}\right)\left[\int_{0}^{N}N_{s}\;e^{N_{s}}dN_{s}-\int_{x_{0}}^{x_{1}}N_{s}\;e^{N_{s}}dN_{s}\right]$$ (26)

Range *x*_0_ to *x*_1_ defines the interval for which the delays are negligible or delays are well below the threshold value. Clearly, network instability increases with increase in randomness and network size, or it can be deduced that the network stability increases with decrease in randomness and network size/genome size. Hence, the Lemma-2 holds.

***Theorem—1*** *In a fully connected network, with increase in value of mapping and sequencing, stability increases consistently causing delays to be negligible i.e. if genome delays G_d_ are given as functional dependency over N_is_ such that*

$$G_{d}=f\left(N_{is}\right),$$

*then with increase in network coordination, G_d_ limits to functional dependency on mapping M_p_ and genome content G_c_ i.e.*

$$G_{d}=f\left(M_{p},G_{c}\right)$$

***Proof:*** This theorem can be proved using Lemma-1 and Lemma-2. For maximum network connections, mapping *M*_*p*_ is defined as fully connected graph such that

$$M_{p}=\frac{N_{s}\left(N_{s}-1\right)}{2}$$ (27)

For networks comprising of larger number of nodes as in the case of the proposed approach which comprise of multiple networks, *M*_*p*_ > >*N*_*s*_. Also, with increase in sequencing *S*_*q*_, network randomness drops constantly causing network stability to increase abruptly depending only upon the network size and mapping nodes. Thus, for larger values of *N*_*s*_ and fully connected network links, *M*_*p*_ causes *N*_*is*_ to converge its countability only on *G*_*c*_ and *M*_*p*_ neglecting dependency over *N*_*s*_. Therefore, for a fully connected network, network instability will be defined as:

$$N_{is}=\left(1-\frac{1}{M_{p}\;G_{c}}\right)$$ (28)

Using Lemma-2, in a network, delays can be defined as functional dependency on network instability such that

$$G_{d}\propto \frac{1}{N_{is}}$$ (29)

Using Eqs (28) and (29), it can be deduced that *G*_*d*_ = *f*(*M*_*p*_, *G*_*c*_) for very large values of *N*_*s*_.

### 3.5 Network Functioning

The proposed model aims at collaborating multiple networks via UA centric data forwarding allowing the formation of delay tolerant network. In the proposed network formation, relays are selected from the multi-grouped networks which are capable of providing connectivity without many delays. For improved connectivity, UA acts as an intermediate between any two networks with different genome identifier. The maximum tendency is towards the selection of relays that have lower instability rate and have less randomness count for the overall network size.

**Algorithm 1** Selection of Intra-Relays

**Require**: *N*_*s*_, So ←Source, De ←Destination

**Ensure**: Network ← Up, Genome ←Active

 set *G*_*c*_

 path = route[]

 **while** (path != true) **do**

  i = 1

  **while** (i ≤ *N*_*s*_) **do**

   broadcast(HELLO)

   route = route[So,De]

   *N*_*f*_

   **if** (*N*_*t*_(*So*) == *N*_*t*_(*De*)) **then**

    Compute *N*_*r*_

    **if** $(N_{is}\leq N_{is}^{TH}$) **then**

     route[] ← N(i)

     **if** ($G_{d}\leq G_{d}^{TH}$) **then**

      map(i-1,i)

      path ← route[i-1,i]

      increment *G*_*c*_

      increment *S*_*q*_

      *N*(*i*) = *S*_*q*_

     **else**

      rebroadcast()

     **end if**

    **else**

     retrace()

    **end if**

   **else**

    j = *N*_*t*_(*De*)

    path ← UA_communicate(*i*, *j*, *G*_*c*_, *S*_*q*_)

   **end if**

   i = i+1

  **end while**

  iterate

 **end while**

**Algorithm 2** Selection of Inter-Relays and UA Mapping

**Require**: call UA_communicate(*i*, *j*, *G*_*c*_, *S*_*q*_)

**Ensure**: *i*, *j*, *G*_*c*_, *S*_*q*_ ← active

 fetch *G*_*c*_

 UA_counter = *G*_*c*_

 **while** path! = complete **do**

  identify N(j)

  **if** (N(j) == found) **then**

   mark *N*_*f*_

   allocate *S*_*q*_

   coordinates ← GPS(N(j))

   identify UA(coordinates)

   N(UA)

   **if** ((*LoS*(*UA*), *De*) == *available*) **then**

    route[]← N(j), N(UA)

    check *N*_*is*_ and *G*_*d*_

    **if** (satisfied) **then**

     path ← route[UA,j]

     increment *UA*_*counter*

     increment *S*_*q*_

    **else**

     rebroadcast()

    **end if**

   **else**

    identify alternate node

   **end if**

  **else**

   No path, retract

  **end if**

  iterate

 **end while**

 return path

In most of the cases, relays are selected from the network to which source and the destination belong; thus, limiting the counter effects of other underlying networks on the current operational networks. But in some cases, three or more network nodes have to be considered due to non-availability of direct connectivity or lack of line of sight facility with the above maneuvering UA. This type of network formation is termed as hybrid-genome networking that has to be efficient and capable of converging in accordance with Lemma-1 and Lemma-2. Two major subroutines are used to identify the nodes which will be mapped together to form a fully-reliable delay tolerant network.

The first subroutine aims at a selection of intra-genome mapping. The complexity of this mapping is as low as “HELLO” intervals used in normal path findings. The second subroutine is called by the first one in the case of non-availability of connectivity with the destination node and requirement of UA as intermediate. This subroutine maps the node from one network with the node of another network via UA. Thus, it can be noticed that in the case of inter-network formation, one of the chromosomes in both directional network function must be that of a UA. Algorithm 1, and Algorithm 2 present the steps depicting the above-explained procedure for the selection of appropriate relays and mapping UA in the case of inter-network message exchanges. Algorithm 1 uses the genome communication set up defined over *N*_*is*_ to select the appropriate route between the network nodes. This algorithm uses the incremental policy to find the optimal path between the network nodes based on the active genomes.

In the next step, Algorithm 2 provides an inter-relay facility with mapping of UA to different network nodes. This algorithm uses the GPS and the line of sight (LOS) provisioning to check for availability of nodes based on their sequence number *S*_*q*_. This algorithm also considers the location of the source and the destination for inter-network connectivity.

## 4 Performance Evaluation

The proposed GDTN formation was analyzed in two parts. The first evaluation was carried out on real-time testbed using live UA. The testbed was customized to support the intra- and inter-network connectivity between the different networks. 50 static nodes were considered each using the in-house network connectivity for data transmission maneuvered by three UAs. In the second part, in order to evaluate the performance of the proposed model, the state of the art approaches were simulated and compared with the proposed model. The simulation environment used to compare the performance was inspired by the similar settings as that of the real-time testbed. The simulation setup was developed in *Matlab*^*TM*^ with traffic generated from a module coded in C#, and the existing approaches were observed from ONE Simulator [35]. The analysis presented in the sub-section depicts the performance of the proposed and the existing delay tolerant models over the hybrid network formations.

### 4.1 Real-time Testbed

**Fig 3:**
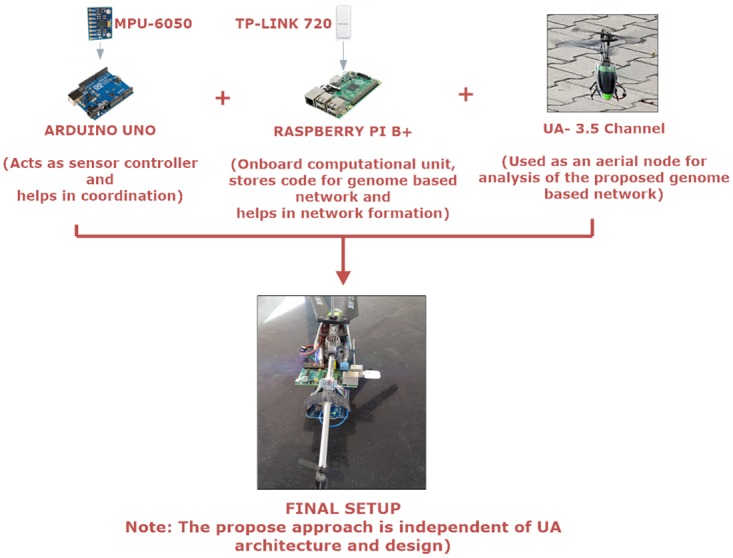
UA Configurations for Testbed.

**Fig 4:**
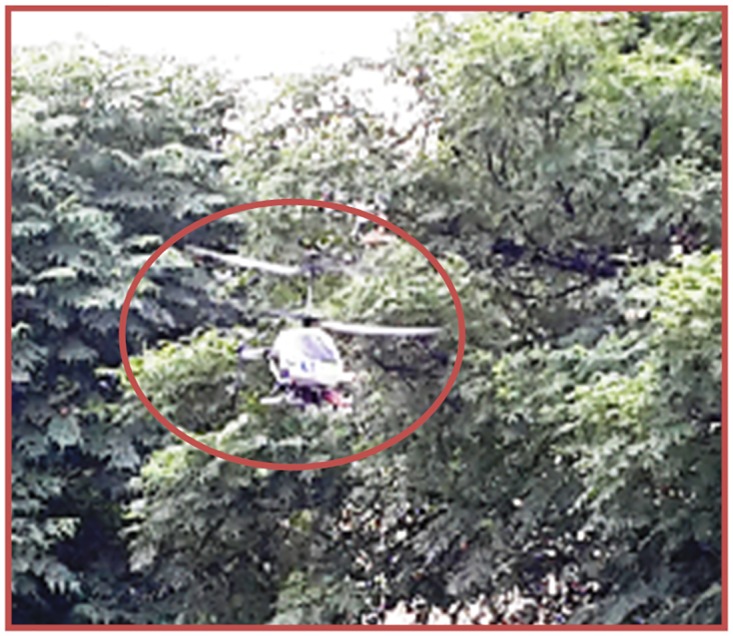
UA-1 in action.

**Fig 5:**
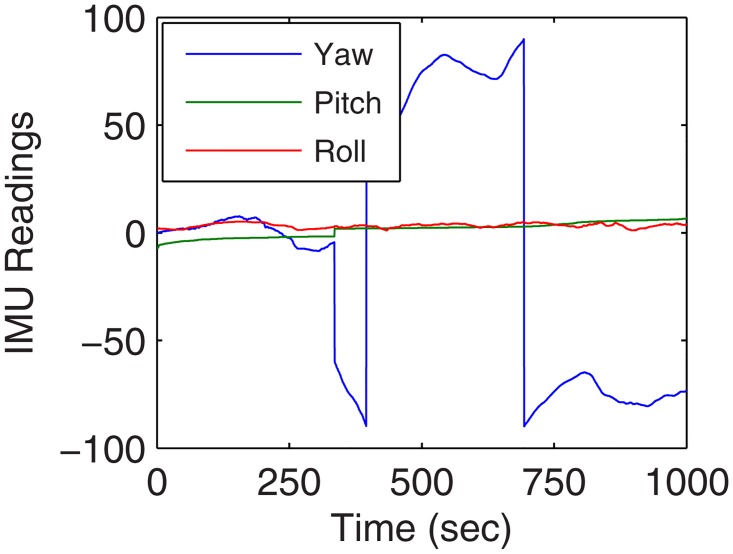
IMU readings for UA-1 vs. Operational Time.

Real-time analysis was performed over testbed which comprises of hybrid nodes capable of forming multiple networks in an area of 1000x1000*m*^2^. The area used for analysis comprised of both static and mobile nodes which were maneuvered using custom waypoint based 3 UA. The configuration of UA used for analysis of the proposed approach is presented in Fig 3. Various other configurations for testbed are shown in Table 1. Fig 4 shows a pictorial view of the UA-1 in action, Fig 5 presents the IMU readings traced for its movement during performance analysis. Graphical traces were recorded for analyzing the performance of the proposed hybrid network formation.

**Table 1:** Testbed Configurations.

Parameter	Value
UA	3
UA-Channel	3.5
Area	1000mx1000m
Virtual Nodes (each machine)	2–3
Network Size	100 nodes
Flight Time	35 mins
Connectivity Available	20 mins
Recording Time	17 mins
UA Type	Rotor Wing
Radio Frequency	2.5GHz
Onboard Process	Raspberry Pi(Model B+)
Sensor Controller	Arduion Uno
IMU	MPU 6050
Wireless Adapter	TP-link 720
Data Analyzer	Dell Precision T15610
GPS	GPS-10710

**Fig 6:**
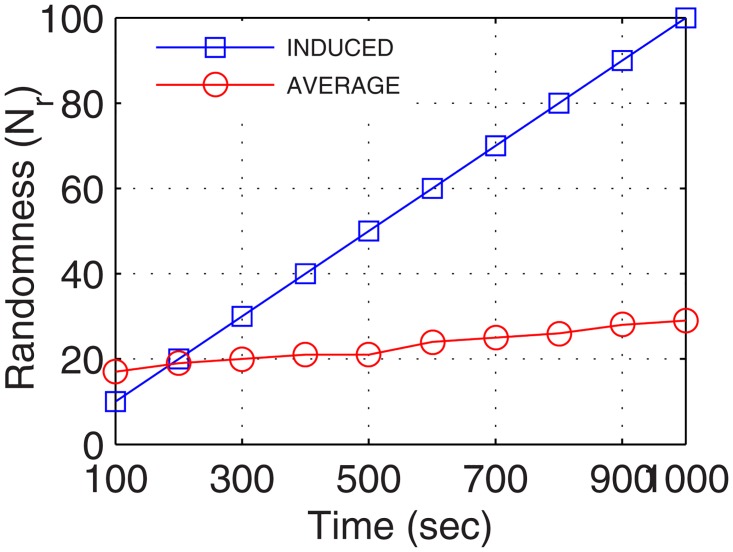
Randomness vs. Operational Time.

**Fig 7:**
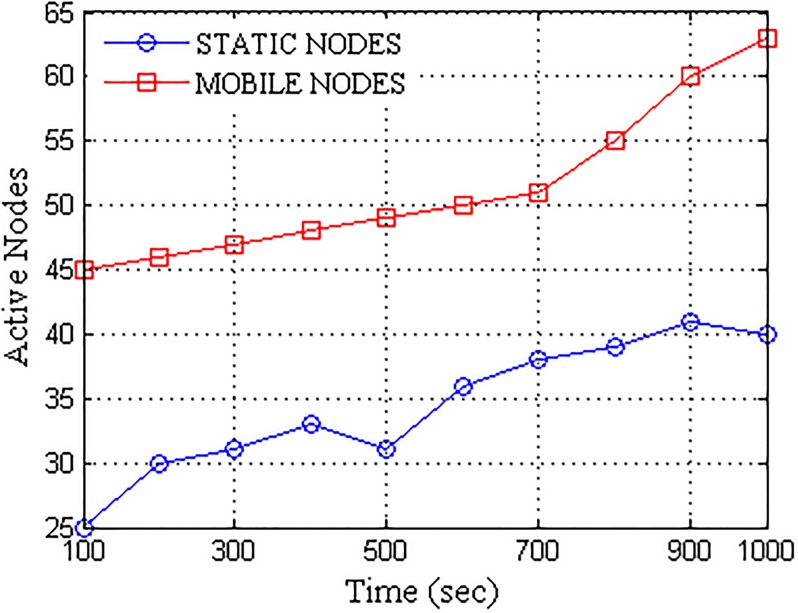
Active nodes vs. Operational Time.

Fig 6 presents the randomness considered and induced to test the robustness of the proposed network formation. For analysis, the induced randomness increased linearly with respect to the operational time. The induced randomness allowed replication of almost complete network with a set of non-configurable nodes to perform a robust analysis. With network randomness increasing linearly, active nodes comprising of both static, as well as the dynamic nodes, increased with respect to the operational time. The plot for the number of active nodes traced with variation in time is shown in Fig 7.

**Fig 8:**
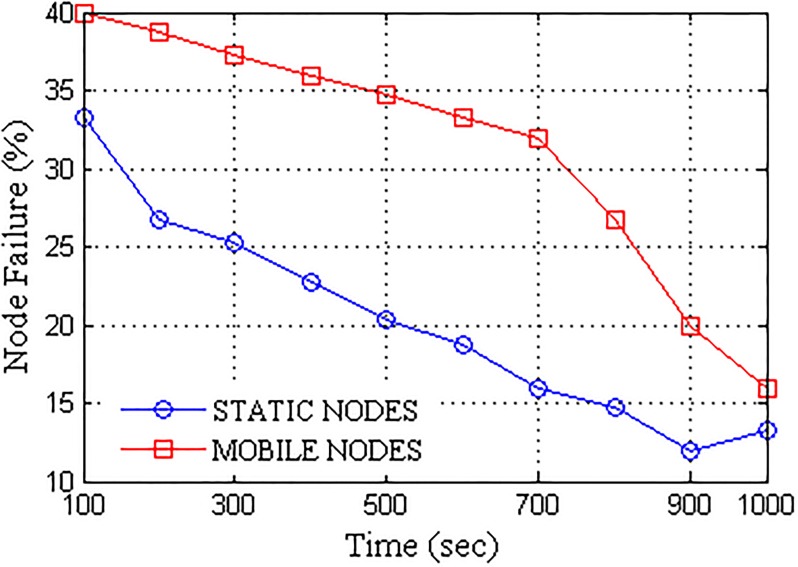
Node Failures vs. Operational Time.

**Fig 9:**
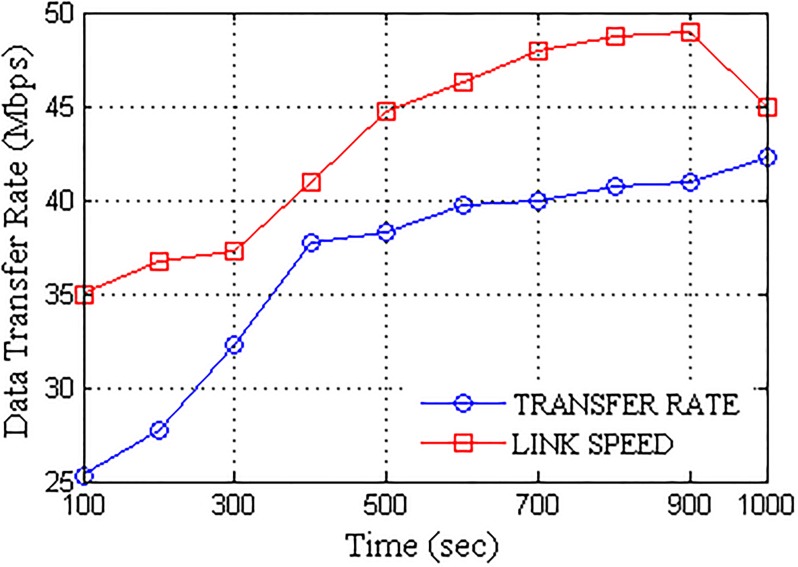
Data Rate vs. Operational Time.

**Fig 10:**
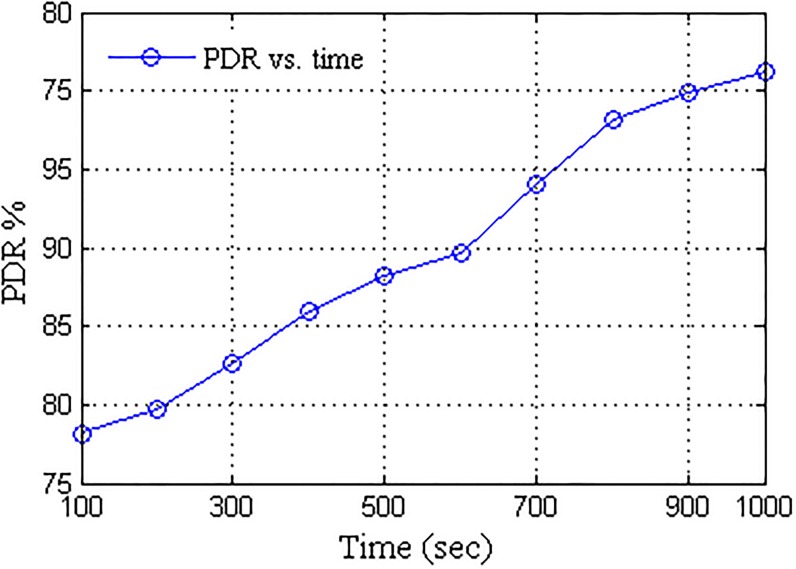
PDR vs. Operational Time.

Further, the network was subjected to the dynamic failure of nodes in order to replicate an actual delay occurring condition. The percentage of failures computed over the active nodes induced in the network are presented in Fig 8. A network with transfer rate closer to actual link speed is considered to be efficient. The proposed network formation with UA acting as a pivot for data transfers provided higher transfer rate which converged towards the maximum link speed with the passage of time. Better localization of nodes is attained after certain time stamps which result in improved data transfer rate. Despite the randomness and less number of nodes available for customized transmission as per the scenario, the proposed approach provides better connectivity, thus, the overall transfer rate increases with the time as the network randomness gradually decreases. The comparison for available link speed and achieved transfer rate is presented in Fig 9. With prolonged connectivity, the randomness of the network is overcome by the active nodes which in turn improves the transfer rate. A network is considered to be efficient if it offers better performance in terms of data transfers. Packet delivery ratio (PDR) provides the successful percentage of data delivered to the destination. More the PDR, better is the connectivity, thus, efficient is the network. With gradual decrease in the network randomness, a number of active nodes increases so as the network transfer rate, which in turn decreases the network failure rate, thus, improving the PDR with the passage of time. Fig 10 presents the packet delivery ratio (PDR) attained by the UA pivoted delay tolerant network. Further, the network was analyzed for major parameters required to test the performance of DTNs, namely, overheads and delays. Overheads account over transmission time error that hinders the data transmission whereas delays account over propagation, transmission, processing and queuing time.

**Fig 11:**
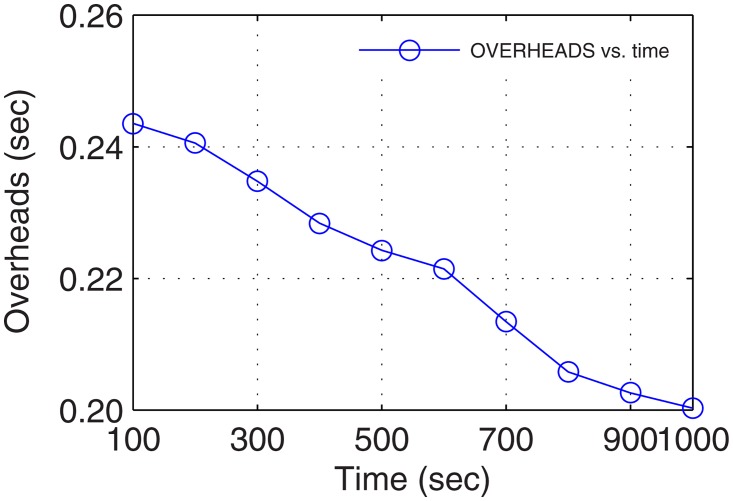
Overheads vs. Operational Time.

**Fig 12:**
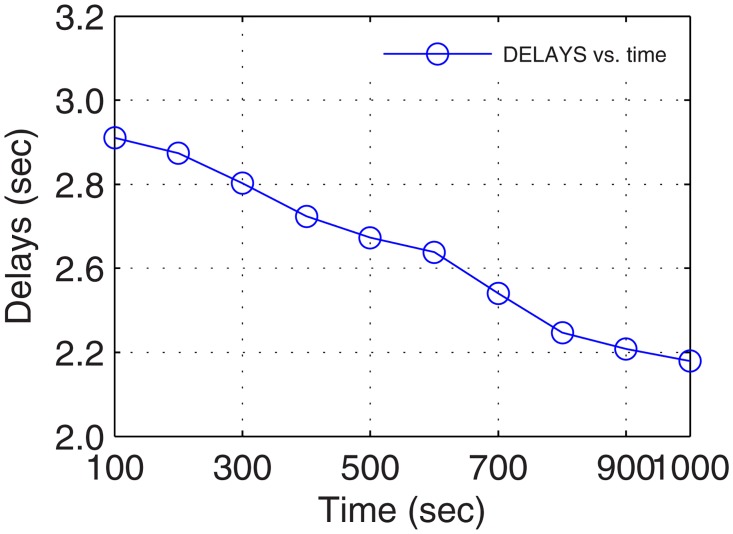
Delays vs. Operational Time.

In the real-time environment, delays were traced in seconds as all the considered networks are prescribed for this standard. However, the delays recorded after analysis for 17 minutes of connectivity with above maneuvering UA were sufficiently low proving the worth of forming UA-oriented GDTN. Plots for overheads and delays are presented in Figs 11 and 12, respectively. The delays and overheads presented in these plots show a continuous decrease over the connectivity time. This is because of the increase in the number of active nodes and decrease in the network randomness.

### 4.2 Simulations

In order to validate the proposed model, comparative analysis was carried against with existing state-of-the-art DTN models, namely: Bubble [7], Epidemic [8], Prophet [9] and greedy forwarding. For analysis, the network simulations were configured to provide operability for all these models. The configurations and parameters are shown in Table 2. A total of 50 simulation runs were performed to trace the comparison between the considered approaches.

**Table 2:** Simulation Configurations.

Parameter	Value
Area	1000x1000sq. m
Network Size	100 nodes
UA	1,5,10,15
Traffic Type	CBR/VBR
Packet Size	1024 bytes
Window Size	20 packets
Propagation Radio Model(Ground)	Two Ray Ground
Mobility Model(Ground)	Random way point
Mobility Model(Aerial)	Custom way point
Pause Time	2.0s
Buffer	10–100
Radio Range	500m
UA Control Range	1000m
Simulation Time	1000 s

The proposed work is evaluated with existing approaches using both real-time testbed and simulation environment, in terms of:

- **Packet Delivery Ratio (PDR):** The ratio of successfully delivering packets to the total packets transmitted during DTN formation using UA as a relay.
- **Overheads:** Overheads account for transmission time error that hinders data transmission and includes the time for excessive and recursive computations.
- **Network Delays:** Network delays are the sum of the propagational, transmission, queuing, and processing time. Delays check for the time laps in providing services to the end users.
- **Convergence Rate:** Network convergence rate is defined as the ratio of the time taken by the network to stabilize and total connectivity time.
- **Cost of Convergence:** Cost of convergence is computed as the product of ratio of convergence rate and number of iterations with number of nodes
- **Network Randomness:** Network randomness is the percentage of nodes which are non-configurable and do not follow any network rules. Also, it accounts for the network nodes which do not participate in relaying.
- **Network Fairness:** Network fairness accounts for determining the fair share of resources between the network nodes.

#### 4.2.1 Packet Delivery Ratio (PDR)

**Fig 13:**
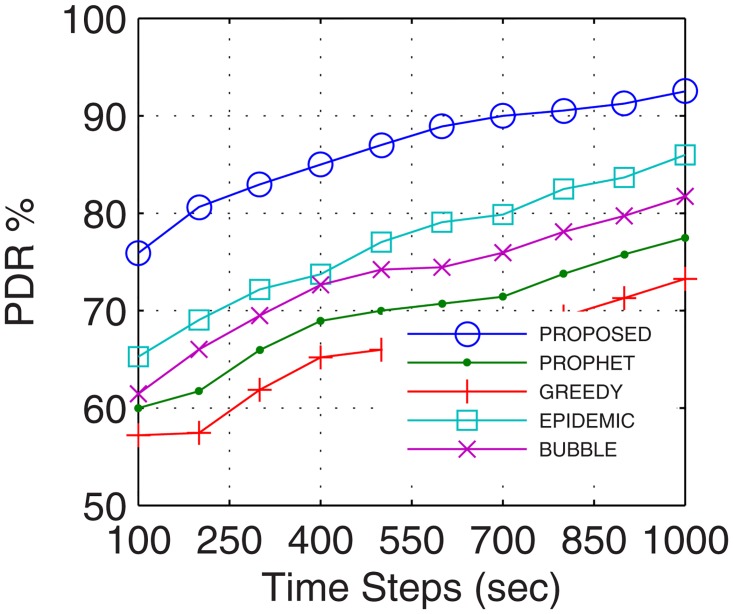
PDR vs. Time.

**Fig 14:**
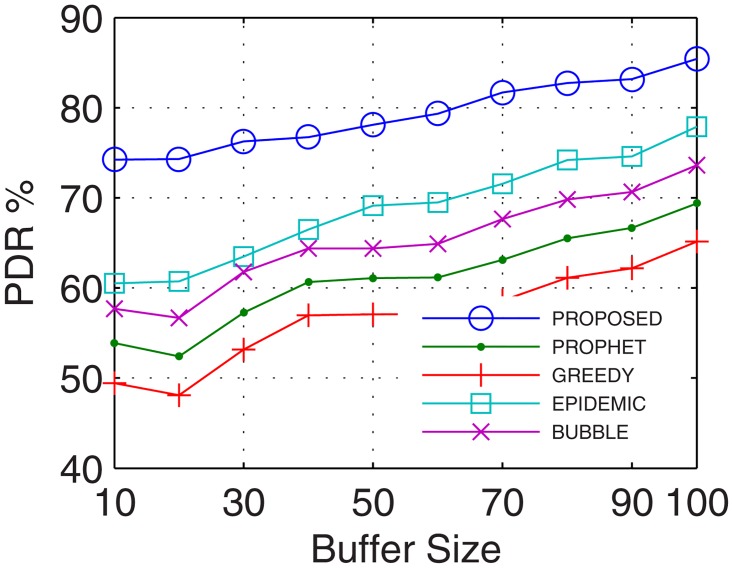
PDR vs. Buffer.

**Fig 15:**
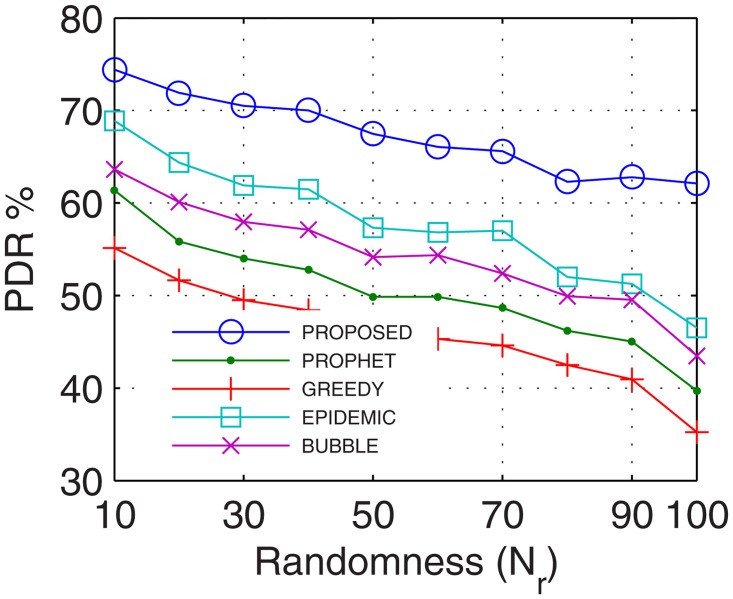
PDR vs. Randomness.

**Fig 16:**
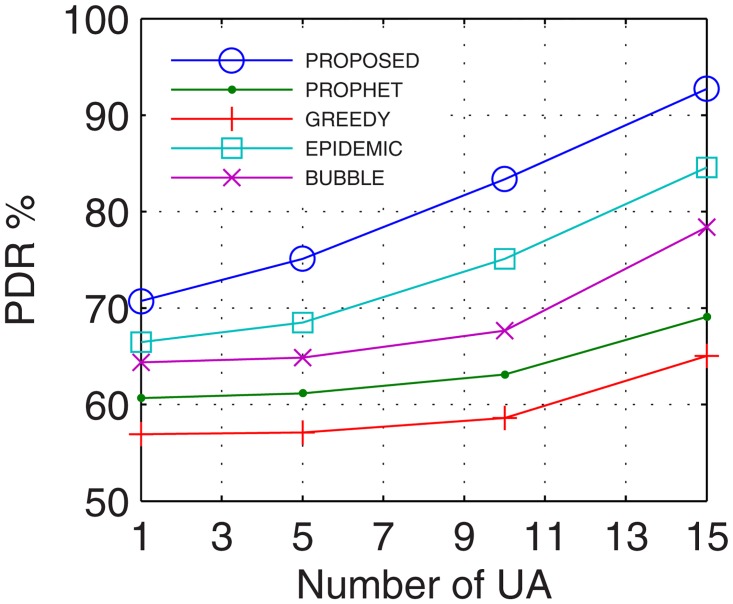
PDR vs. UA.

Packet delivery ratio accounts for the successful delivery of data to the end users. With prolonged connectivity, the number of active nodes increases by using the proposed GDTN approach. Further, GDTN decreases the network randomness by providing better stability and availability of inter-UAs for relaying. This provides multiple paths between the end users which increase the overall data rate, and thus, improves the PDR. Analyses show that the proposed GDTN model showed improvement of 11%, 15%, 20.5%, and 25.3% (shown in Fig 13) for delivery ratio vs. time in comparison with the epidemic, bubble, prophet, and greedy approaches, respectively. For variation in the buffer, percentage improvement was 13%, 17%, 22.4%, and 27.80% (shown in Fig 14), for variation in randomness, percentage improvement recorded was 14%, 19.29%, 25.14%, and 31.7% (shown in Fig 15), and for variation in number of UA, the percentage improvement traced was 16.11%, 19.7%, 34.9% and 36.6% (shown in Fig 16) against the epidemic, bubble, prophet and greedy approaches, respectively.

#### 4.2.2 Network Overheads

**Fig 17:**
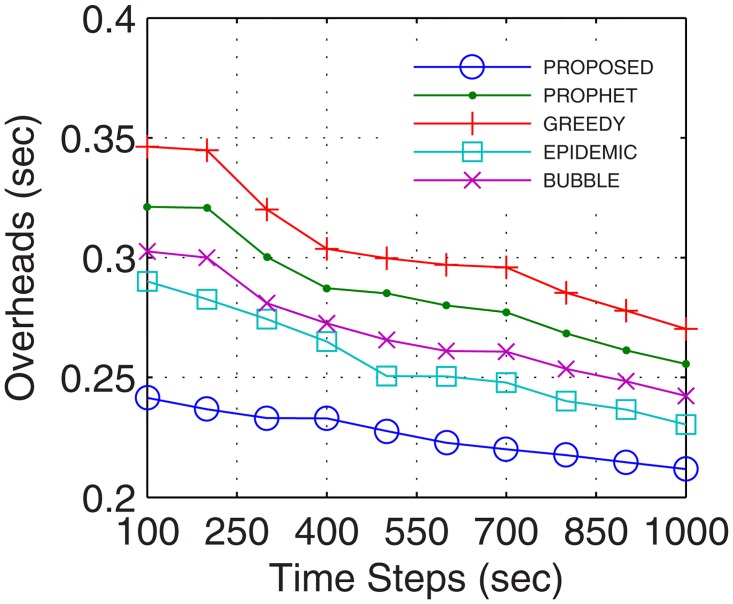
Overheads vs. Time.

**Fig 18:**
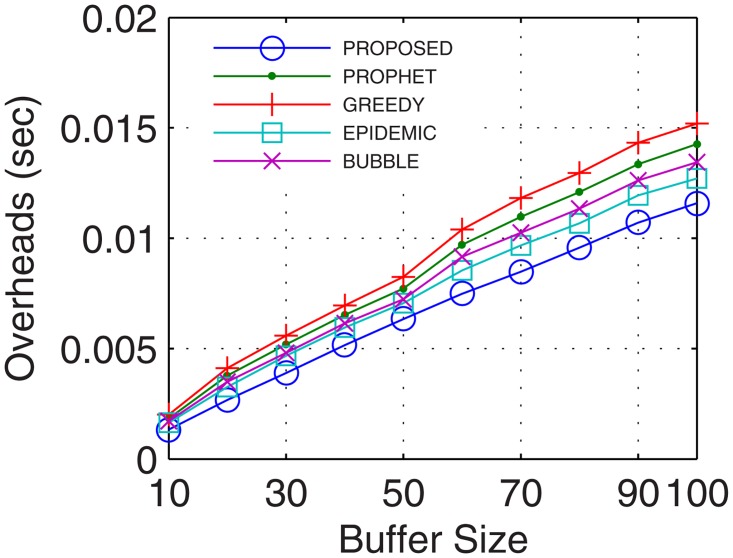
Overheads vs. Buffer.

**Fig 19:**
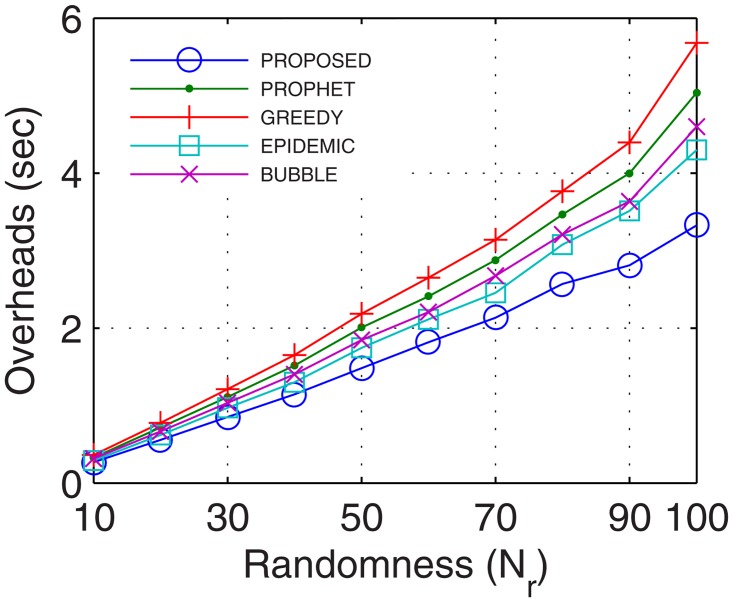
Overheads vs. Randomness.

**Fig 20:**
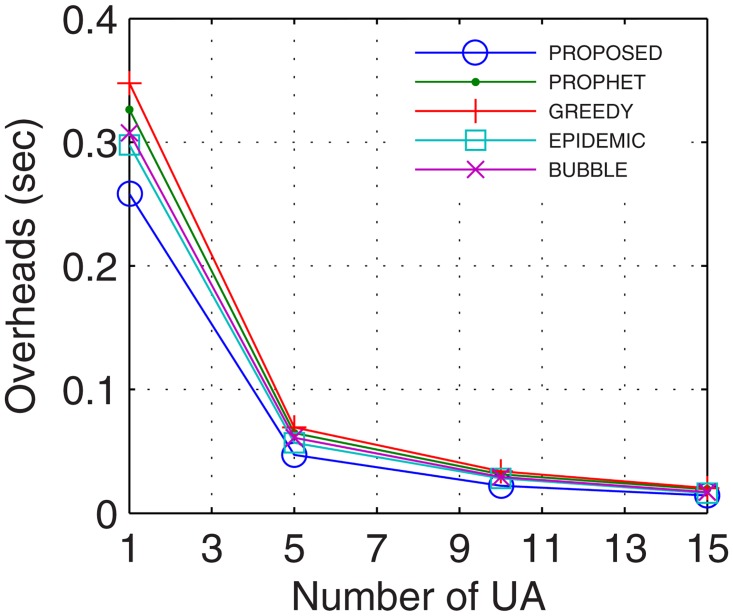
Overheads vs. UA.

Network overheads account for the transmission time error that hinders the data transmission and also includes the time for excessive and recursive computations. With more nodes actively involved in transmission, the packet header increases which increases the network overheads. With stable and continuous connectivity in the proposed GDTN approach, the network overheads followed the supporting trend with respect to the buffer, randomness, connectivity time, and the number of UAs. With an increase in the buffer, more computations are required to maintain the flow. But, these computations are low enough that lesser overheads are observed in the proposed approach as compared to the existing solutions. Also, with the increase in network randomness, the overheads are bound to increase. With an increase in randomness, the proposed GDTN actively stabilizes the network by the provisioning of inter-UA relays. However, with more UAs and prolonged connectivity, the number of active nodes as well as the path for transfer increases which gradually decreases the overall network overheads. Results for network overheads show that the improvement against epidemic, bubble, prophet, and greedy approaches with respect to time was 12.4%, 15.38%, 21.42%, and 26.6% (shown in Fig 17), respectively; with respect to buffer was 22.8%, 25%, 25.8%, and 35.12% (shown in Fig 18), respectively; with respect to variation in randomness was 16.7%, 25.58%, 31.6%, and 37.9% (shown in Fig 19), respectively; with respect to variation in number of UA was 11.1%, 21.2%, 27.2%, and 27.34% (shown in Fig 20), respectively.

#### 4.2.3 Convergence Rate

**Fig 21:**
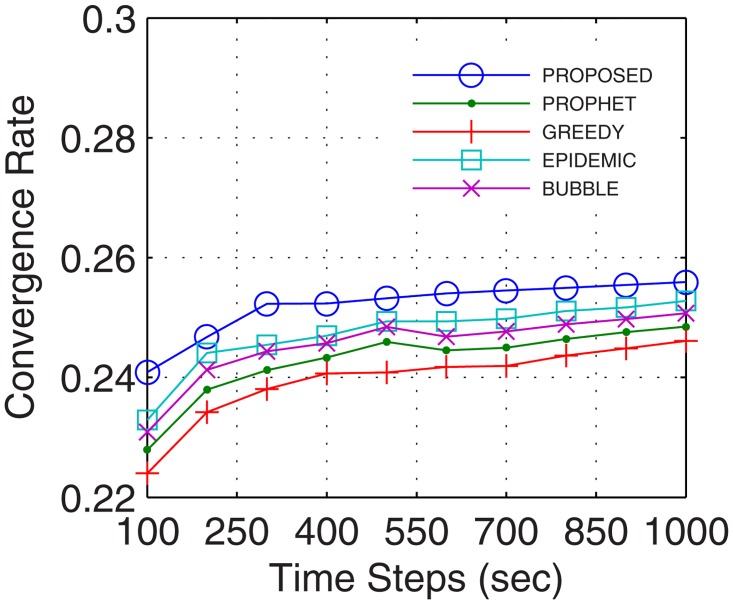
Convergence Rate vs. Time.

**Fig 22:**
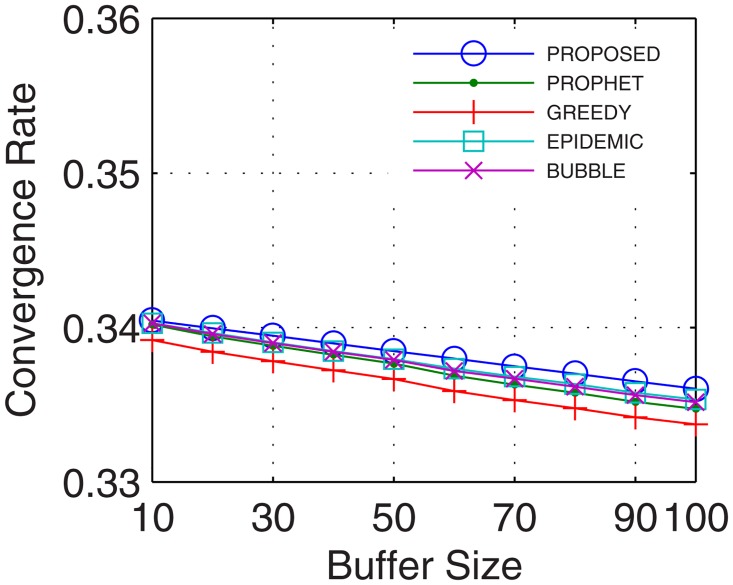
Convergence Rate vs. Buffer.

**Fig 23:**
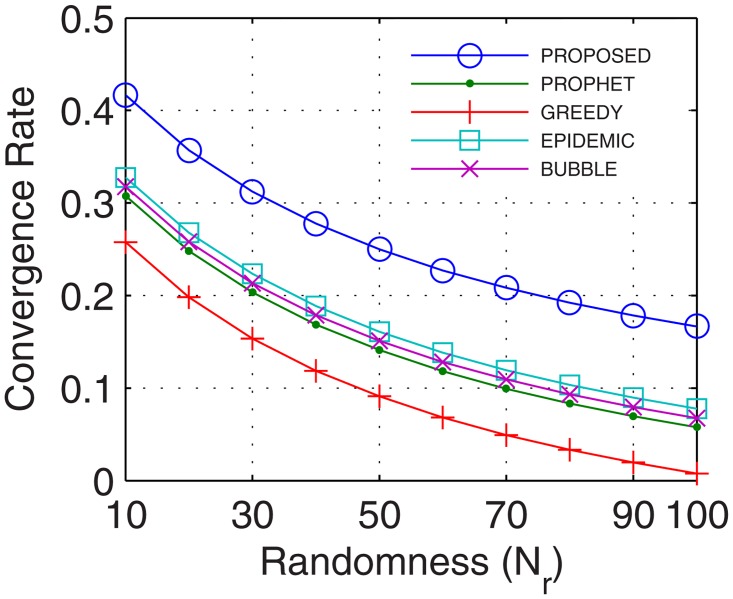
Convergence Rate vs. Randomness.

**Fig 24:**
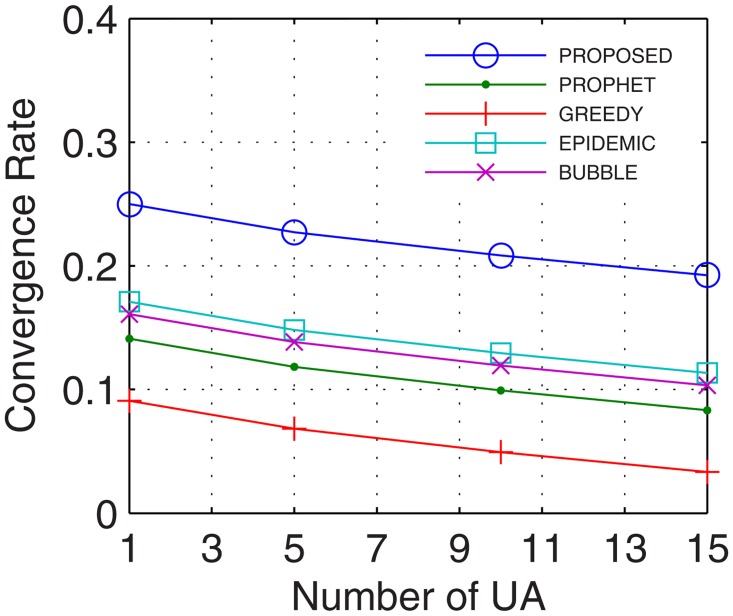
Convergence Rate vs. UA.

Analyses were also traced for convergence rate, which is defined as the ratio of the time taken by the network to stabilize and total connectivity time. With the number of active nodes increasing continuously during the transmission between the end users, the convergence rate improves. Further, with inter-UA support for relaying, continuous flow is attained which quickly stabilizes the network and provides better transfer rate. Results show that the proposed GDTN approach offered better convergence by the use of inter-UA in comparison with the existing approaches. Percentage enhancement for convergence rate in comparison with epidemic, bubble, prophet and greedy with respect to time was 14%, 24.8%, 25.6%, and 25.8% (shown in Fig 21), respectively; with respect to variation in buffer size was 32%, 33.8%, 35.6%, and 36.8% (shown in Fig 22), respectively; with respect to randomness was 36%, 40%, 40.4%, and 43.5% (shown in Fig 23), respectively; with respect to number of UA was 33.3%, 31%, 47.6%, and 71.4% (shown in Fig 24), respectively.

#### 4.2.4 Cost of Convergence

**Fig 25:**
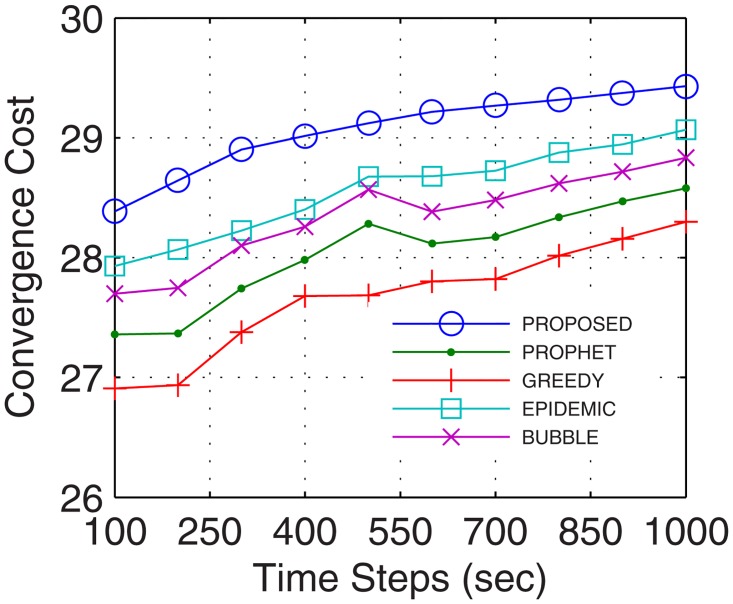
Cost of Convergence vs. Time.

**Fig 26:**
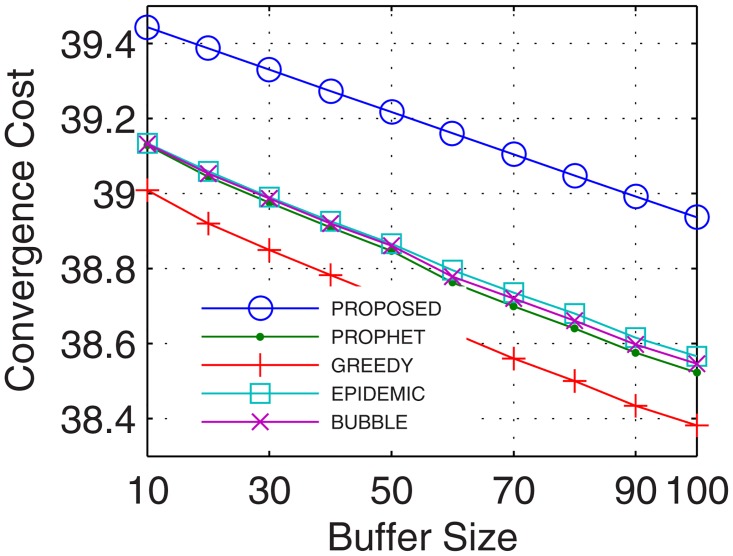
Cost of Convergence vs. Buffer.

**Fig 27:**
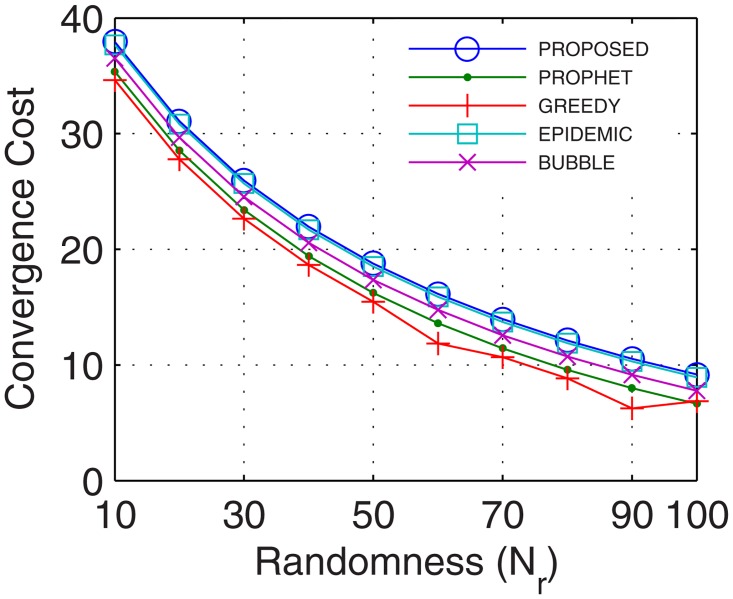
Cost of Convergence vs. Randomness.

**Fig 28:**
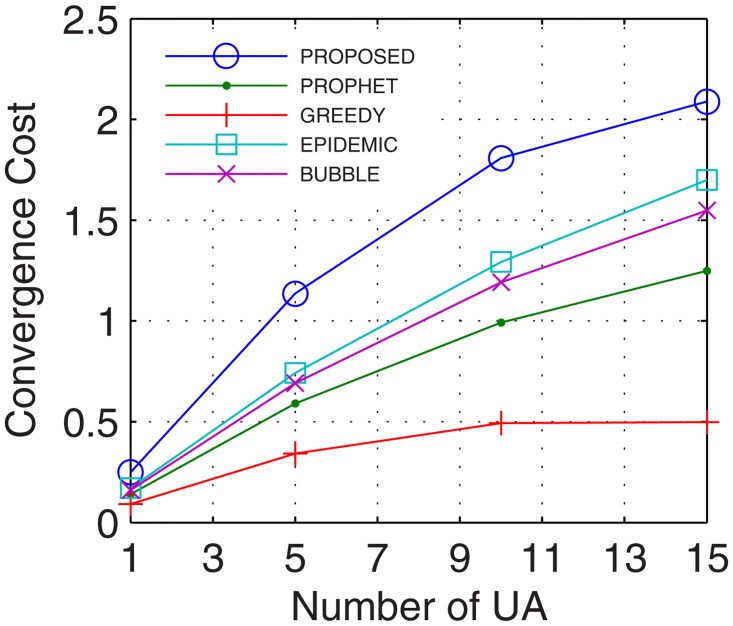
Cost of Convergence vs. UA.

This is the only limitation in the proposed model. In this paper, the cost of convergence is computed as the product of convergence rate and the number of iterations required by the number of nodes. With more nodes, the number of iterations required to stabilize the network increases which increases the cost of convergence. Another reason for the increase in the cost of convergence is the hybrid nature of nodes involved in network formation. With nodes possessing different configurations, the cost of convergence increases as more iterations are required to map the flow between these hybrid nodes. The proposed model utilized, 2%, 12%, 4%, and 20%, more cost in comparison to epidemic over time, buffer, randomness, and the number of UA, respectively. In comparison with bubble, the excessive cost was 2.7%, 12.04%, 6%, and 20.6%, for a time, buffer, randomness, and the number of UA, respectively. Over similar variations, the excessive cost required with respect to the prophet was 2.8%, 12.04%, 12.63%, and 23.3%, and in comparison with the greedy percentage increase in cost was 4.8%, 12.2%, 14.4%, and 23.5%. The plots for convergence cost vs. buffer, randomness, time and number of UA are shown in Figs 25, 26, 27 and 28, respectively. However, with the flow being the priority, and formation of efficient DTNs as the primary task, the cost of convergence can be neglected as the transfer rate attained with an increase in the number of iterations is sufficiently large to make the cost negligible.

#### 4.2.5 Network Delays

**Fig 29:**
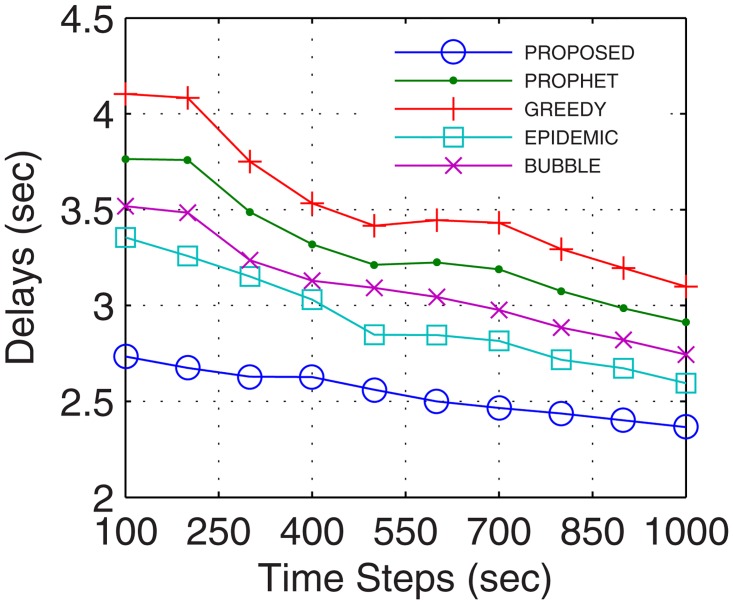
Delays vs. Time.

**Fig 30:**
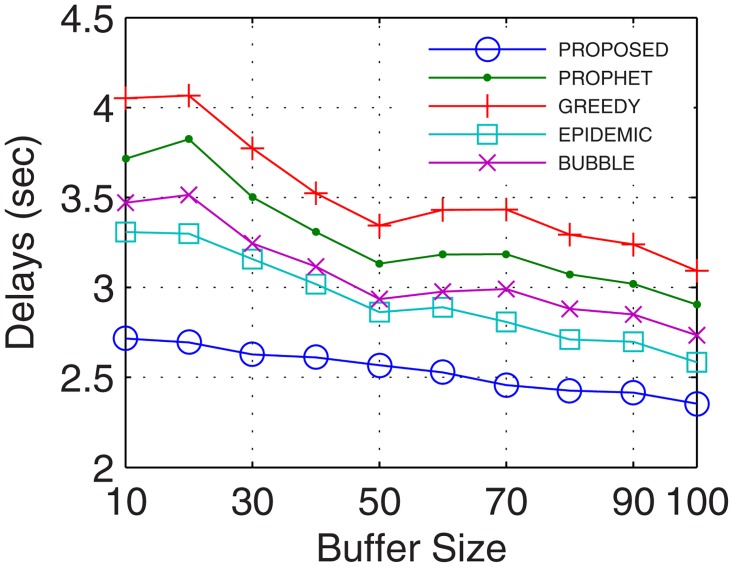
Delays vs. Buffer.

**Fig 31:**
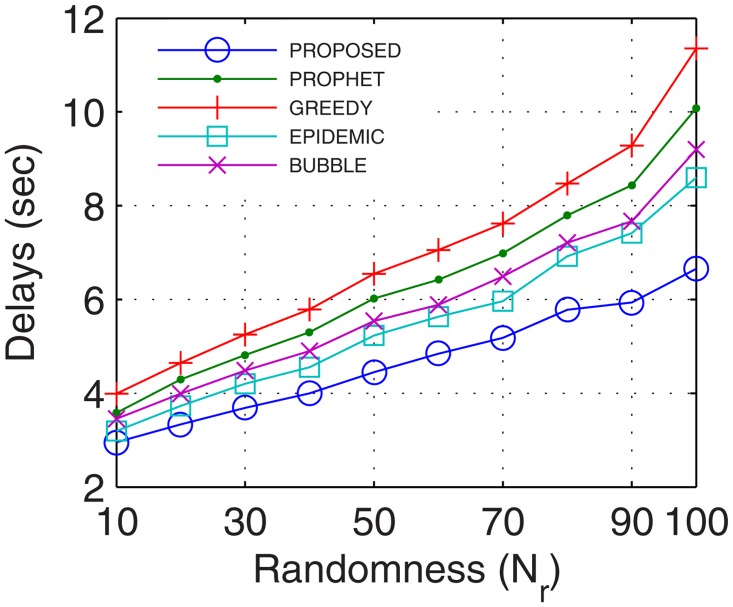
Delays vs. Randomness.

**Fig 32:**
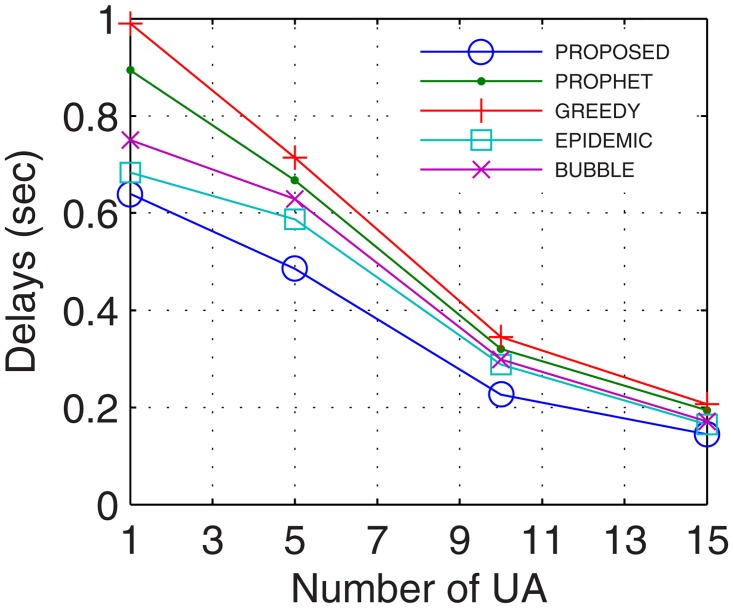
Delays vs. UA.

The most important parameter for analyzing the efficiency of the proposed networks is the delays occurred during normal network operations. Delays account for the transmission, propagational, queueing, and processing time. More the number of nodes, better are the chances for the availability of path between the end users, thus, lower are the delays. With inter-UA support as a relay, the proposed GDTN approach leverages lesser issues in the selection of the path over the active nodes, which improves the connectivity and decreases the delay. Percentage improvement in delays for the proposed model in comparison with the epidemic, bubble, prophet and greedy, with respect to time was 13.7%, 18.5%, 23.7%, and 28.5% (shown in Fig 29), respectively; with variation in buffer size was 13.7%, 23.2%, 24.0%, and 28.5% (shown in Fig 30), respectively; with variation in randomness was 16.9%, 20.4%, 24.6%, and 33.1% (shown in Fig 31), respectively; with variation in number of UA was 10%, 12.6%, 17.6% (shown in Fig 32), and 25.1%, respectively. Analysis proved that the proposed GDTN can provide efficient delay tolerant connectivity between the nodes of the network with different configurations. Improvement in terms of network parameters proves the utility of the proposed model in dynamic network conditions.

#### 4.2.6 Statistical Confidence Intervals

**Fig 33:**
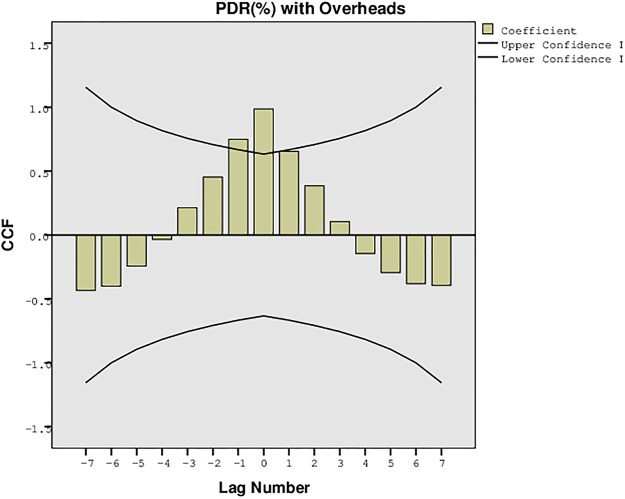
Confidence Plot for PDR (%) with Overheads.

**Fig 34:**
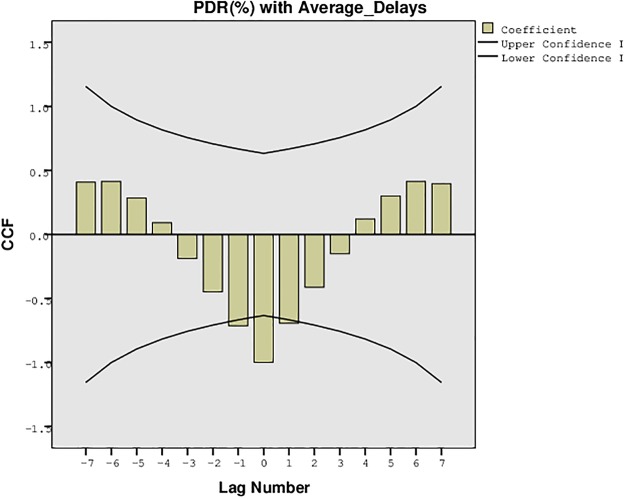
Confidence Plot for PDR (%) with Average Delays.

**Fig 35:**
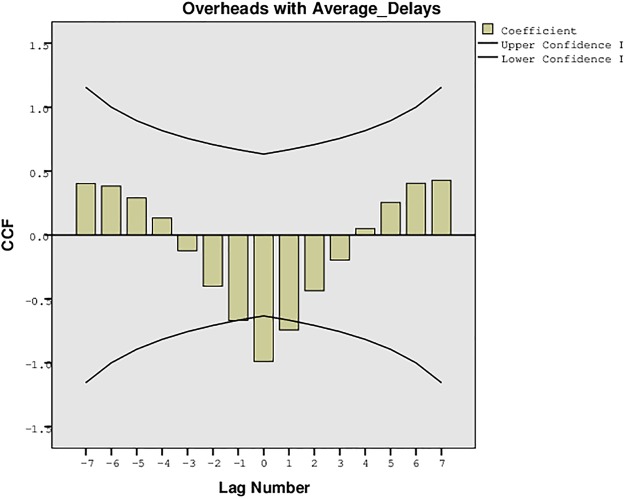
Confidence Plot for Overheads with Average Delays.

The proposed approach for the formation of a delay tolerant network was also evaluated for the statistical confidence intervals to analyze the upper and lower bounds for the PDR, overheads, and the average network delays. These intervals were recorded for the one sample t-test with variation in the buffer size, time, and number of UAs. The results were traced for 95% confidence interval of the difference with lower and upper bounds, mean difference, and the standard deviation as shown in Tables 3, 4, 5 and 6. The results presented in these tables also validate the simulation results recorded for the proposed approach. The confidence interval suggests that the proposed approach is much stable and precise despite the variation of parameters. The variation in the number of UAs has much impact on the performance than the variation in buffer size, time and randomness. These intervals can be further understood from the plots presented in Fig 33, 34 and 35. Fig 33 presents the confidence limit analysis for PDR and overheads with 95% interval limit, Fig 34 presents the confidence limit analysis for PDR and average delays with 95% interval limit, and Fig 35 presents the confidence limit analysis for overheads and average delays with 95% interval limit. These intervals suggest that despite the impact of overheads and average delays, the proposed approach possess high PDR throughout the connectivity time.

**Table 3:** 95% Confidence Interval of the Difference for the Variation in Buffer Size.

Parameter	Lower	Upper	Mean Difference	Std. Deviation
PDR(%)	76.4048619	81.9985581	79.20171000	3.90972409
Overheads	.00425092	.00919612	.006723520	.003456452
Average_Delays	2.4509736	2.6239085	2.53744104	.12087317

**Table 4:** 95% Confidence Interval of the Difference over Simulation Time.

Parameter	Lower	Upper	Mean Difference	Std. Deviation
PDR(%)	85.0415471	90.6061129	87.82383000	3.88936332
Overheads	.2186797	.2330216	.22585063	.01002427
Average_Delays	2.4505702	2.6282236	2.53939691	.12417116

**Table 5:** 95% Confidence Interval of the Difference for the variation in Randomness.

Parameter	Lower	Upper	Mean Difference	Std. Deviation
PDR(%)	64.0283207	70.4489993	67.23866000	4.48774485
Overheads	.9696695	2.4220654	1.69586744	1.01515476
Average_Delays	3.8148	5.5499	4.68237	1.21272

**Table 6:** 95% Confidence Interval of the Difference for the variation in number of UA.

Parameter	Lower	Upper	Mean Difference	Std. Deviation
PDR(%)	74.6603927	96.7896573	85.72502500	6.95353906
Overheads	.0990978	.2698344	.08536829	.11592724
Average_Delays	.0850595	.9449575	.42994901	.32365571

## 5 Comparative Summary, Discussions and Open Issues

Apart from the detailed evaluation presented above, a comparative summary is presented in Table 7, in terms of scalability based on the need for additional network components, support for 5G HetNets, UA assistance, topology management, buffer management, and the quality of experience (QoE) to the end users. The comparative summary indicates whether a particular model has the considered property (or not). This allows identification of extra features which can be scaled in the existing solutions on the basis of the requirement for the particular type of applications. From the existing solutions, the proposed GDTN is capable of efficiently using the UAs in the formation of hybrid delay tolerant network. Topology management is the basic requirement of a delay tolerant network; thus, is provided in most of the approaches. Buffer management is one of the crucial aspect missing in most of the approaches and should be incorporated for efficient network functioning. Scalability is another aspect which needs to be considered along with the support for hybrid nodes such as UAs. Apart from all these aspects, one of the most important parameters is the QoE. A network with high QoE is always better in terms of coverage and capacity. Thus, it is necessary to focus on this parameter while developing and deploying approaches for delay tolerant networking. Apart from these, other key issues yet to be resolved include energy efficient buffer management, network energy conservation in DTNs, sleep mode facility in DTNs, efficient packet selection and congestion window management and node energy preservation. It is also necessary to identify the type of traffic and their sources for better localization in DTNs. These aspects collectively allows formation of an efficient hybrid DTN which can provide high-quality service and vast range of applications to end users in the heterogeneous 5G environment. The detail results and analyses reports are provided in the supplementary files, see S1 Files.

**Table 7:** Comparison of the State-of-art Models For Delay Tolerant Network Formations: High (↑), Medium (↔), Low (↓).

Approach	Author (Year)	Ideology	Scalability	Support for HetNets	UA Support	Topology Management	Buffer Management	QoE
Bubble	Hui et al. (2011) [7]	Social based forwarding	↑	✔	✘	✔	✘	↔
Epidemic	Vahdat et al. (2000) [8]	Routing for partially connected networks	↑	✔	✘	✔	✘	↑
Prophet	Lindgren et al. (2003) [9]	Probabilistic routing	↑	✔	✘	✔	✘	↔
Autonomous Drone	Schoeneich et al. (2016) [19]	Autonomous message ferry	↓	✘	✔	✔	✔	↓
MPAR	You et al. (2015) [22]	Pattern aware routing	↑	✔	✘	✔	✔	↔
CASPaR	Stewart et al. (2016) [23]	Congestion proof shortest path selection	↔	✔	✘	✔	✔	↔
MORA	Burns et al. (2008) [24]	Robotic assistance	↔	✘	✘	✔	✔	↔
SAW	Spyropoulos et al. (2008) [25]	Spray and Wait	↔	✔	✘	✔	✘	↔
MaxProp	Burgess et al. (2006) [26]	Vehicular disruption tolerant networking	↔	✔	✘	✔	✘	↔
Proposed GDTN	You et al.	Genome-based network formation	↑	✔	✔	✔	✔	↑

## 6 Conclusion

Hybrid networks are capable of addressing existing network challenges such as fault-tolerance, continuous connectivity, node failures, and speedy transmission. In this paper, a novel model was presented for incorporating multiple networks together using a centric UA network which acts as a pivot and regulates the traffic between the different networks. The proposed model is inspired from the biological aspect of genomes which provide efficient strategies of chromosome mapping for node connectivity. To demonstrate the utility of the proposed model, we evaluated the model using real-time testbed and using simulations between the proposed GDTN scheme and the epidemic, bubble, prophet and greedy approaches. The evaluations demonstrated that GDTN is capable of providing delay aloof data delivery; thus, forming an efficient delay tolerant hybrid network.

Future work includes extending the proposed model to provide energy efficient management of the buffer, reduced packet redundancy, and utilizing the partial flooding mechanisms in the UA-assisted formations. This will result in higher data rates and better coverage to users in 5G heterogeneous delay tolerant networks.

## Supporting Information

S1 Files Results and Traces. The supplementary material provided with this manuscript contains data set for statistical outputs, hardware traces, comparison results, and the files to regenerate the similar results.
(ZIP)

## References

[1] Yilmaz ON, Wijting C, Lundén P, Hämäläinen J. Optimized mobile connectivity for bandwidth-hungry, delay-tolerant cloud services toward 5G. In: 11th International Symposium on Wireless Communications Systems (ISWCS): IEEE; 2014. pp. 6–10.

[2] Prasad A, Lundén P, Moisio M, Uusitalo MA, Li Z. Efficient mobility and traffic management for delay tolerant cloud data in 5G networks. In: 26th Annual International Symposium on Personal, Indoor, and Mobile Radio Communications (PIMRC): IEEE; 2015. pp. 1740–1745.

[3] Sharma V, Kumar R. A Cooperative Network Framework for Multi-UAV Guided Ground Ad Hoc Networks. Journal of Intelligent & Robotic Systems. 2015;77(3–4):629–652. 10.1007/s10846-014-0091-0

[4] Soares VN, Farahmand F, Rodrigues JJ. A layered architecture for vehicular delay-tolerant networks. In: IEEE Symposium on Computers and Communications; 2009. pp. 122–127.

[5] Jones EP, Li L, Schmidtke JK, Ward PA. Practical routing in delay-tolerant networks. IEEE Trans on Mobile Computing. 2007;6(8):943–959. 10.1109/TMC.2007.1016

[6] Han B, Hui P, Kumar VA, Marathe MV, Shao J, Srinivasan A. Mobile data offloading through opportunistic communications and social participation. IEEE Trans on Mobile Computing. 2012;11(5):821–834. 10.1109/TMC.2011.101

[7] Hui P, Crowcroft J, Yoneki E. Bubble rap: Social-based forwarding in delay-tolerant networks. IEEE Trans on Mobile Computing. 2011;10(11):1576–1589. 10.1109/TMC.2010.246

[8] Vahdat A, Becker D. Epidemic routing for partially connected ad hoc networks. Technical Report CS-200006, Duke University; 2000.

[9] Lindgren A, Doria A, Schelén O. Probabilistic routing in intermittently connected networks. ACM SIGMOBILE mobile computing and communications review. 2003;7(3):19–20. 10.1155/2014/865071

[10] Soares VN, Rodrigues JJ, Farahmand F. GeoSpray: A geographic routing protocol for vehicular delay-tolerant networks. Information Fusion. 2014;15:102–113. 10.1016/j.inffus.2011.11.003

[11] Gil-Castineira F, Gonzalez-Castano FJ, Franck L. Extending vehicular CAN fieldbuses with delay-tolerant networks. IEEE Trans on Industrial Electronics. 2008;55(9):3307–3314. 10.1109/TIE.2008.927972

[12] Chen J, Cao X, Cheng P, Xiao Y, Sun Y. Distributed collaborative control for industrial automation with wireless sensor and actuator networks. IEEE Trans on Industrial Electronics. 2010;57(12):4219–4230. 10.1109/TIE.2010.2043038

[13] Rubinstein MG, Ben Abdesslem F, De Amorim MD, Cavalcanti SR, Santos RAD, Costa LHMK, et al Measuring the capacity of in-car to in-car vehicular networks. IEEE Communications Magazine. 2009;47(11):128–136. 10.1109/MCOM.2009.5307476

[14] Pereira PR, Casaca A, Rodrigues JJ, Soares VN, Triay J, Cervelló-Pastor C. From delay-tolerant networks to vehicular delay-tolerant networks. IEEE Communications Surveys & Tutorials. 2012;14(4):1166–1182. 10.3390/s16101567

[15] Åkerberg J, Gidlund M, Björkman M. Future research challenges in wireless sensor and actuator networks targeting industrial automation. In: Industrial Informatics (INDIN): IEEE; 2011. pp. 410–415.

[16] Shuai Z, Zhang H, Wang J, Li J, Ouyang M. Combined AFS and DYC control of four-wheel-independent-drive electric vehicles over CAN network with time-varying delays. IEEE Trans on Vehicular Technology. 2014;63(2):591–602. 10.1109/TVT.2013.2279843

[17] Rao W, Zhao K, Zhang Y, Hui P, Tarkoma S. Towards maximizing timely content delivery in delay tolerant networks. IEEE Trans on Mobile Computing. 2015;14(4):755–769. 10.1109/TMC.2014.2330296

[18] Khanesar MA, Kaynak O, Yin S, Gao H. Adaptive Indirect Fuzzy Sliding Mode Controller for Networked Control Systems Subject to Time-Varying Network-Induced Time Delay. IEEE Trans on Fuzzy Systems. 2015;23(1):205–214. 10.1109/TFUZZ.2014.2362549

[19] Schoeneich RO, Golański M, Krok B, Czermiński P. Autonomous drone for delay-tolerant networks in indoor applications. International Journal of Distributed Sensor Networks. 2016;12(8):1550147716662755 10.1177/1550147716662755

[20] Tian C, Ci L, Cheng B, Li X. A 3D Location-Based Energy Aware Routing Protocol in Delay Tolerant Networks. In: IEEE DASC; 2014. pp. 485–490.

[21] Yu C, Tu Z, Yao D, Lu F, Jin H. Probabilistic routing algorithm based on contact duration and message redundancy in delay tolerant network. International Journal of Communication Systems. 2015; 10.1002/dac.3030

[22] You L, Li J, Wei C, Hu L. MPAR: A movement pattern-aware optimal routing for social delay tolerant networks. Ad Hoc Networks. 2015;24:228–249. 10.1016/j.adhoc.2014.09.004

[23] Stewart MF, Kannan R, Dvir A, Krishnamachari B. CASPaR: Congestion avoidance shortest path routing for delay tolerant networks. In: 2016 International Conference on Computing, Networking and Communications (ICNC). IEEE; 2016. pp. 1–5.

[24] Burns B, Brock O, Levine BN. MORA routing and capacity building in disruption-tolerant networks. Ad hoc networks. 2008;6(4):600–620. 10.1016/j.adhoc.2007.05.002

[25] Spyropoulos T, Psounis K, Raghavendra CS. Efficient routing in intermittently connected mobile networks: the multiple-copy case. IEEE/ACM transactions on networking. 2008;16(1):77–90. 10.1155/2014/865071

[26] Burgess J, Gallagher B, Jensen D, Levine BN. MaxProp: Routing for Vehicle-Based Disruption-Tolerant Networks. In: INFOCOM. 2006;6 pp. 1–11. 10.3390/s16040436

[27] Ramasamy S, Sabatini R. A unified approach to cooperative and non-cooperative Sense-and-Avoid. In: ICUAS; 2015. pp. 765–773.

[28] Sharma V, Kumar R. An opportunistic cross layer design for efficient service dissemination over flying ad hoc networks (FANETs). In: ICECS; 2015. pp. 1551–1557.

[29] Sharma V, Kumar R, Patiala P. Service-Oriented Middleware for Multi-UAV Guided Ad Hoc Networks. IT CoNvergence PRActice (INPRA). 2014;2(3):24–33.

[30] Ridley M. Genome. 1st ed Harper and Collins; 2000.

[31] Ludwig A, Schmidt KD. Gauss–Markov loss prediction in a linear model. In: Casualty Actuarial Society E-Forum; Fall 2010.

[32] Tenne D, Singh T. Characterizing performance of *α*-*β*-*γ* filters. IEEE Transactions on Aerospace and Electronic Systems. 2002;38(3):1072–1087.

[33] Jain R. The art of computer systems performance analysis. 9788126519057. 1st ed John Wiley & Sons; 2008.

[34] Figueiras J, Frattasi S. Mobile positioning and tracking: from conventional to cooperative techniques. 1st ed John Wiley & Sons; 2011 10.1002/9780470663035

[35] Keränen A, Ott J, Kärkkäinen T. The ONE Simulator for DTN Protocol Evaluation. In: SIMUTools’09: Proceedings of the 2nd International Conference on Simulation Tools and Techniques. New York USA: ICST; 2009. pp. 1–10.

