# Supplementary material for: GDTN: Genome-Based Delay Tolerant Network Formation in Heterogeneous 5G Using Inter-UA Collaboration
Source: PLoS One. 2016 Dec 14;11(12):e0167913. doi: 10.1371/journal.pone.0167913 (PMC5156398; doi:10.1371/journal.pone.0167913)
Supplement: S1 Files — The supplementary material provided with this manuscript contains data set for statistical outputs, hardware traces, comparison results, and the files to regenerate the similar results. (ZIP) [file pone.0167913.s001.zip › Detailed_results_datasets/OUTPUT1.doc]

One-Sample Test	
	Test Value = 0                                       	
	t	df	Sig. (2-tailed)	Mean Difference	
Buffer_Size	5.745	9	.000	55.00000	
PDR(%)	64.060	9	.000	79.20171000	
Overheads	6.151	9	.000	.006723520	
Average_Delays	66.384	9	.000	2.53744104	

One-Sample Test	
	Test Value = 0                                       	
	95% Confidence Interval of the Difference	
	Lower	Upper	
Buffer_Size	33.3415	76.6585	
PDR(%)	76.4048619	81.9985581	
Overheads	.00425092	.00919612	
Average_Delays	2.4509736	2.6239085	
