# Supplementary material for: GDTN: Genome-Based Delay Tolerant Network Formation in Heterogeneous 5G Using Inter-UA Collaboration
Source: PLoS One. 2016 Dec 14;11(12):e0167913. doi: 10.1371/journal.pone.0167913 (PMC5156398; doi:10.1371/journal.pone.0167913)
Supplement: S1 Files — The supplementary material provided with this manuscript contains data set for statistical outputs, hardware traces, comparison results, and the files to regenerate the similar results. (ZIP) [file pone.0167913.s001.zip › Detailed_results_datasets/OUTPUT3.doc]

One-Sample Test	
	Test Value = 0                                       	
	t	df	Sig. (2-tailed)	Mean Difference	
PDR(%)	71.406	9	.000	87.82383000	
Overheads	71.247	9	.000	.22585063	
Average_Delays	64.671	9	.000	2.53939691	

One-Sample Test	
	Test Value = 0                                       	
	95% Confidence Interval of the Difference	
	Lower	Upper	
PDR(%)	85.0415471	90.6061129	
Overheads	.2186797	.2330216	
Average_Delays	2.4505702	2.6282236	
