# Supplementary material for: GDTN: Genome-Based Delay Tolerant Network Formation in Heterogeneous 5G Using Inter-UA Collaboration
Source: PLoS One. 2016 Dec 14;11(12):e0167913. doi: 10.1371/journal.pone.0167913 (PMC5156398; doi:10.1371/journal.pone.0167913)
Supplement: S1 Files — The supplementary material provided with this manuscript contains data set for statistical outputs, hardware traces, comparison results, and the files to regenerate the similar results. (ZIP) [file pone.0167913.s001.zip › Detailed_results_datasets/OUTPUT4.doc]

One-Sample Statistics	
	N	Mean	Std. Deviation	Std. Error Mean	
PDR(%)	10	87.8238300	3.88936332	1.22992467	
Overheads	10	.2258506	.01002427	.00316995	
Average_Delays	10	2.5393969	.12417116	.03926637	
