# Supplementary material for: GDTN: Genome-Based Delay Tolerant Network Formation in Heterogeneous 5G Using Inter-UA Collaboration
Source: PLoS One. 2016 Dec 14;11(12):e0167913. doi: 10.1371/journal.pone.0167913 (PMC5156398; doi:10.1371/journal.pone.0167913)
Supplement: S1 Files — The supplementary material provided with this manuscript contains data set for statistical outputs, hardware traces, comparison results, and the files to regenerate the similar results. (ZIP) [file pone.0167913.s001.zip › Detailed_results_datasets/OUTPUT6.doc]

One-Sample Statistics	
	N	Mean	Std. Deviation	Std. Error Mean	
PDR(%)	10	67.2386600	4.48774485	1.41914953	
Overheads	10	1.6958674	1.01515476	.32102012	
Average_Delays	10	4.6824	1.21272	.38350	
