# Supplementary material for: GDTN: Genome-Based Delay Tolerant Network Formation in Heterogeneous 5G Using Inter-UA Collaboration
Source: PLoS One. 2016 Dec 14;11(12):e0167913. doi: 10.1371/journal.pone.0167913 (PMC5156398; doi:10.1371/journal.pone.0167913)
Supplement: S1 Files — The supplementary material provided with this manuscript contains data set for statistical outputs, hardware traces, comparison results, and the files to regenerate the similar results. (ZIP) [file pone.0167913.s001.zip › Detailed_results_datasets/OUTPUT7.doc]

One-Sample Test	
	Test Value = 0                                       	
	t	df	Sig. (2-tailed)	Mean Difference	
PDR(%)	24.657	3	.000	85.72502500	
Overheads	1.473	3	.237	.08536829	
Average_Delays	2.657	3	.077	.42994901	

One-Sample Test	
	Test Value = 0                                       	
	95% Confidence Interval of the Difference	
	Lower	Upper	
PDR(%)	74.6603927	96.7896573	
Overheads	-.0990978	.2698344	
Average_Delays	-.0850595	.9449575	
