# Supplementary material for: GDTN: Genome-Based Delay Tolerant Network Formation in Heterogeneous 5G Using Inter-UA Collaboration
Source: PLoS One. 2016 Dec 14;11(12):e0167913. doi: 10.1371/journal.pone.0167913 (PMC5156398; doi:10.1371/journal.pone.0167913)
Supplement: S1 Files — The supplementary material provided with this manuscript contains data set for statistical outputs, hardware traces, comparison results, and the files to regenerate the similar results. (ZIP) [file pone.0167913.s001.zip › Detailed_results_datasets/OUTPUT8.doc]

One-Sample Statistics	
	N	Mean	Std. Deviation	Std. Error Mean	
PDR(%)	4	85.7250250	6.95353906	3.47676953	
Overheads	4	.0853683	.11592724	.05796362	
Average_Delays	4	.4299490	.32365571	.16182786	
